# The Revelation of Continuously Organized, Co-Overexpressed Protein-Coding Genes with Roles in Cellular Communications in Breast Cancer

**DOI:** 10.3390/cells11233806

**Published:** 2022-11-28

**Authors:** Aswathy Mary Paul, Revikumar Amjesh, Bijesh George, Deivendran Sankaran, Oleta A. Sandiford, Pranela Rameshwar, Madhavan Radhakrishna Pillai, Rakesh Kumar

**Affiliations:** 1 Cancer Research Program, Rajiv Gandhi Centre for Biotechnology, Thiruvananthapuram 695014, India; 2 PhD Program, Manipal Academy of Higher Education, Manipal 576104, India; 3 Department of Medicine-Hematology and Oncology, Rutgers New Jersey Medical School, Newark, NJ 07103, USA; 4 Cancer Research Institute, Himalayan Institute of Medical Sciences, Swami Rama Himalayan University, Dehradun 248016, India; 5 Department of Human and Molecular Genetics, School of Medicine, Virginia Commonwealth University, Richmond, VA 23298, USA

**Keywords:** breast cancer, computational biology, polygenic nature, coregulation, shared mechanisms of gene expression, transcriptome, emerging pathways, cancer progression

## Abstract

Many human cancers, including breast cancer, are polygenic and involve the co-dysregulation of multiple regulatory molecules and pathways. Though the overexpression of genes and amplified chromosomal regions have been closely linked in breast cancer, the notion of the co-upregulation of genes at a single locus remains poorly described. Here, we describe the co-overexpression of 34 continuously organized protein-coding genes with diverse functions at 8q.24.3(143437655-144326919) in breast and other cancer types, the CanCord34 genes. In total, 10 out of 34 genes have not been reported to be overexpressed in breast cancer. Interestingly, the overexpression of CanCord34 genes is not necessarily associated with genomic amplification and is independent of hormonal or HER2 status in breast cancer. CanCord34 genes exhibit diverse known and predicted functions, including enzymatic activities, cell viability, multipotency, cancer stem cells, and secretory activities, including extracellular vesicles. The co-overexpression of 33 of the CanCord34 genes in a multivariant analysis was correlated with poor survival among patients with breast cancer. The analysis of the genome-wide RNAi functional screening, cell dependency fitness, and breast cancer stem cell databases indicated that three diverse overexpressed CanCord34 genes, including a component of spliceosome PUF60, a component of exosome complex EXOSC4, and a ribosomal biogenesis factor BOP1, shared roles in cell viability, cell fitness, and stem cell phenotypes. In addition, 17 of the CanCord34 genes were found in the microvesicles (MVs) secreted from the mesenchymal stem cells that were primed with MDA-MB-231 breast cancer cells. Since these MVs were important in the chemoresistance and dedifferentiation of breast cancer cells into cancer stem cells, these findings highlight the significance of the CanCord34 genes in cellular communications. In brief, the persistent co-overexpression of CanCord34 genes with diverse functions can lead to the dysregulation of complementary functions in breast cancer. In brief, the present study provides new insights into the polygenic nature of breast cancer and opens new research avenues for basic, preclinical, and therapeutic studies in human cancer.

## 1. Introduction

Breast cancer, being a heterogenous disease, often involves the dysregulation of multiple molecules, pathways, and cellular processes. Though some cancers are driven by mutant genes and addicted to specific pathways [1,2], the pathobiology of most cancer types, including breast cancer, is polygenic [2,3,4,5]. The approaches involving an immense focus on a single gene or protein have been very fruitful in revealing the governing principles and underlying mechanisms of candidate gene expression, its upstream regulators, downstream effectors, associated regulatory interactome, and resulting functions. Such approaches were/are essential for reaching the current stage of knowledge about the genomic and molecular basis of dysregulated cancerous pathways and will continue to be important for our ability as cancer biologists to unearth new aspects of molecules of interest. However, it is now clear that in addition to a single gene/protein approach, we may also wish to intensify our effort to better understand the polygenic nature of breast cancer (as well as many other cancer types) and explore broader scientific questions, seeking polygenic solutions. Examples of such questions might include, but are not limited to, the principles of the coordinated expression of co-overexpressed genes, orderly sequential versus simultaneous dysregulation of cellular homeostasis, multi-dimensional signal-dependent regulation of gene expression, etc. In general, a better understanding of the co-upregulation of gene expression and resulting functions would provide new insights into cancer cells’ inner workings and the tools for improved diagnostic and therapeutic avenues. 

The chromosomal amplification of multiple loci represents one of the most prevalent genomic dysregulations in human cancer, accounting for the co-overexpression of genes within amplicons and activating adjacent genes, cancer hotspots, risk loci, etc. [6,7,8,9]. A large body of previous work has established that the amplification and co-amplification of chromosome 8q21-24 and its sub-regions represent some of the most common copy number dysregulations in breast and other epithelial tumors, such as ovarian, colorectal, prostate cancer, etc. [7,8,9,10,11,12,13,14]. The 8q21-24 region also contains functional enhancer elements [10] and several pan gene sets shared by many cancer types [8,12,13,14,15]. The 8q21-24 region homes overexpressed oncogenes, such as MYC, heat shock factor 1 (HSF1), and associated cancer signatures [9,16]. This region also contains several overexpressed amplicons, which are associated with early relapse, chemoresistance, distant metastasis, the disease progression of breast cancer, and the driver genes with a role in the transformation and cell survival [14,15,17,18,19]. The 8q24 region also includes the enhancer elements and epigenetic hotspots with roles in the long-range regulation of gene expression via chromatin looping with MYC and other transforming genes [9,19]. These and other examples of copy number aberrations have solidified the notion of co-dysregulated genes and gene signatures and facilitated the clustering of cancers into sub-classes, etc. [20]. For example, a 54-kb region in between the cellular communication network factor 3 (CCN3 or NOV) and ectonucleotide pyrophosphatase/phosphodiesterase 2 (ENPP2) genes on chromosome 8q24 has been suggested to be associated with the development of breast cancer [20] and co-expression gene patterns during colon cancer metastasis [21]. The amplified region contains co-localized PAN cancer gene signatures of UTP23 and SHARPIN gene signatures [6], and it has enabled the clustering of 21 human cancer types into seven cross-cancer gene signatures based on coregulated genes [22,23]. In brief, an improved understanding of the role and significance of the coordinated overexpression of cancer-relevant genes has emerged as an integral part of our effort to further delineate the polygenic nature of breast cancer. 

At the molecular level, the dysregulation of regulatory pathways and their associated functions in cancer cells is believed to be driven, in part, by the co-overexpression of genes or gene sets, possibly through shared mechanism(s). For example, the dysregulation of critical biologic processes relevant to cellular homeostasis (i.e., proliferation, inflammation, energy metabolism, chromatin and cytoskeleton remodeling, protein translation and immune response, etc.) are shared by several cancer types. To better understand breast cancer’s pathobiology and unearth new facets of the shared dysregulation of cellular pathways, one potential approach is to search for coregulated genes with roles in diverse functions beyond one cancer type. Over the years, we have been pursuing the notion of the polygenic nature of breast cancer [2,3]. In the present discovery study, we attempted to address the co-overexpression of cancer-relevant genes by following a serendipity observation approach and describing the coordinated co-overexpression of a set of 34 continuously organized protein-coding genes with diverse functions in breast cancer. Because of the co-dysregulation of multiple genes with a role in various cancerous processes, our observations suggest that the persistent co-upregulation of such genes can lead to the dysregulation of complementary functions in breast cancer (and in other cancer types where genes are co-upregulated). This, in turn, strengthens the concept that breast cancer, being polygenic and requiring the coordinated dysregulation of cancerous pathways, may best be addressed through polygenic approaches.

## 2. Materials and Methods

### 2.1. Gene Expression Analysis

The gene expression datasets used in our study were downloaded from the cBioPortal for Cancer Genomics [24,25], the University of California Santa Cruz (UCSC) Xena browser [26], and publicly available RNA sequencing or microarray databases. The provisional datasets, named under the Cancer Genome Atlas (TCGA) Firehose Legacy, were used for TCGA breast invasive carcinoma (BRCA) and other cancer types, including TCGA cervical cancer (CESC), TCGA esophageal cancer (ESCA), TCGA head and neck cancer (HNSC), TCGA lower-grade glioma (LGG), TCGA lung adenocarcinoma (LUAD), TCGA ovarian cancer (OV), TCGA prostate cancer (PRAD), TCGA endometrioid cancer (UCEC), and TCGA colon and rectal cancer (COADREAD). We downloaded and analyzed the z-score files for each cancer type while keeping +2 and −2 as the cut-offs for the upregulation and downregulation, respectively. These genes were used to evaluate the expression of the enriched genes of interest. Our analyses of breast cancer classified the data into different subtypes as per the patient’s clinical information and PAM50 status. Samples without any subtype information were excluded from the study. 

The expression patterns observed in the patient sample were also examined in breast cancer cell lines using Broad’s CCLE dataset and other breast cancer datasets [27]. Using the cBioPortal data, we also examined the expression of CanCord34 genes in the Molecular Taxonomy of Breast Cancer International Consortium (METABRIC) [28,29,30]. Comparisons between the tumor and matched normal samples were performed using the data taken from the UCSC Xena browser. The normalized files at level 3 (*n* = 1218) were downloaded for breast invasive carcinoma. The expression values are presented as the log2 (x + 1) transformed RSEM normalized count. The gene expression data were also utilized to classify the data into two groups based on the levels of CanCord34 expression. The data were classified using a k-means classification algorithm with k = 2, using the patterns of the test genes [31], and plotted as a boxplot using the ggplot() function package in the R Bioconductor. The barrier of the non-availability of normal samples for many cancer types in cBioPortal was overcome by examining the normal levels of the CanCord34 genes in the RNA-seq of the tissues and a mixture of 16 tissues (Illumina Body Map) deposited in the ArrayExpress database under the accession number E-MTAB-513 (https://www.ebi.ac.uk/arrayexpress/ experiments/E-MTAB-513/ accessed on 6 February 2019). The TNBC microarray data in the GEO database (https://www.ncbi.nlm.nih.gov/geo/ accessed on 6 February 2019) under the accession numbers GSE31519 [32], GSE58812 [33], and GSE83937 [34] were also curated by downloading them using the GEOquery [35] package of R Bioconductor, followed by their pre-processing and normalization using the RMA method [36]. Subsequently, the normalized expression files were used to compare the TNBC/NORMAL/non-TNBC samples. All the analyzed gene expression datasets were also used for the construction of heatmaps, as needed, using the heatmap algorithm of R Bioconductor and the heatmap algorithm of Gene Pattern [31]. 

The expression of the CanCord34 genes was also analyzed at the protein level using the curated proteogenomic data from the article of Mertins et al. [37]. As it was essential to evaluate the functional importance of the CanCord34 genes, using the same set of 77 patient datasets, we also compared the expression of the CanCord34 genes at the levels of the mRNAs and proteins in the same patient. 

### 2.2. CanCord34 Oncoprint

A consolidated gene alteration profile combining CNV and mRNA expression was directly evaluated using the cBioPortal platform [24,25]. For each cancer type, we selected the putative copy number alterations using the GISTIC and mRNA z-scores compared to their levels in diploid samples (RNA Seq V2 RSEM). The resulting outcome provided an overall profile of the alteration rate for the test genes using the BRCA, CESC, ESCA, HNSC, LGG, LUAD, OV, PRAD, UCEC, and COADREAD.

### 2.3. Statistical Evaluation of Expression

We employed the Wilcoxon test to evaluate the significance of the differences observed between the normal and tumor groups and between two groups in the cancer types of interest. The analysis was executed using the R function Wilcox.test, and we calculated the *p*-value to evaluate the significance. The percentage of alterations for each test gene was directly derived from the cBioPortal site.

### 2.4. Correlation and Network Analyses

To assess the global correlation of the genes in the context of the CanCord34 genes, we used the z-score expression files and calculated the Spearman’s rank correlation value to identify the gene clusters of interest, while the correlation between the CanCord34 genes was computed using the COR method in R Bioconductor. Correlated genes were initially identified keeping a 0.5 value as the positive correlation cut-off, with a *p*-value of less than 0.05. After identifying the correlated genes, we constructed a network using the Cytoscape platform. To evaluate the effect of the amplification of a test gene, we assessed the correlation between the gene and CNV using the cBioPortal platform.

### 2.5. Prognostic Significance

The Kaplan–Meier survival analyses were conducted using the public SurvExpress online tools [38]. To define the prognostic importance of the CanCord34 genes as a group and/or individually, we examined the survival of the patients based on the individual genes and the entire CanCord34 gene set or its subset. An individual assessment of the genes with respect to the survival of patients with breast carcinoma (*n* = 960) was performed. To assess the significance of the CanCord34 genes as a group in different cancer types, we used the TCGA provisional data without any subtype classification. These included: breast invasive carcinoma (*n* = 960), cervical squamous cell carcinoma and endocervical adenocarcinoma (*n* = 191), low-grade gliomas (*n* = 512), lung adenocarcinoma (*n* = 475), colon and rectum adenocarcinoma (*n* = 467), esophageal cancer (*n* = 184), uterus corpus endometrioid carcinoma (*n* = 247), glioblastoma (*n* = 148), head and neck squamous cell carcinoma (*n* = 502), lung squamous cell carcinoma (*n* = 175), pancreatic adenocarcinoma (*n* = 176), skin cutaneous melanoma (*n* = 336), colon adenocarcinoma (*n* = 351), acute myeloid leukemia (*n* = 149), bladder urothelial carcinoma (*n* = 390), adrenocortical carcinoma (*n* = 77), cholangiocarcinoma (*n* = 35), kidney renal clear cell carcinoma (*n* = 415), liver hepatocellular carcinoma (*n* = 361), kidney renal papillary cell carcinoma (*n* = 278), uterine carcinoma (*n* = 57), sarcoma (*n* = 245), stomach adenocarcinoma (*n* = 352), ovarian (*n* = 247), and lymphoid neoplasm diffuse large B-cell lymphomas (*n* = 47). As the CCDC166 gene could not be mapped to the concerned SurvExpress datasets, the multivariant survival analyses included the significance of 33 genes from the CanCord34 list. The data were divided into high and low expressions for high- and low-risk groups, and the survival days were measured to censor the data.

### 2.6. Evidence of CanCord34 Genes as Secreted Molecules with a Role in Stemness

We curated the status of the CanCord34 genes as secreted molecules from the Vesiclepedia database [39]. For each gene, we collected information about the class of extracellular vesicles and the method used for the identification. Another set of curation efforts led to the identification of CanCord34’s role in stemness. We first examined the expression of the CanCord34 genes in ALDH+ and CD44+/CD24 low-expressing cells. We obtained the data from the article by Colacino et al. [40]. The expression values for the CanCord34 genes were compared in the ALDH+ vs. CD24 and CD44 vs. CD22 population datasets. We also curated the RNA-seq dataset from Liu et al. [41], deposited under the accession number SRP100664. The curated dataset was analyzed using an in-house RNA-seq pipeline. Expression values for the CanCord34 genes were extracted and compared between different sub-groups: BCSCs (ALDH+CD24−CD44+), enriched epithelial-like BCSCs (ALDH+non-CD24−CD44+), enriched mesenchymal-like BCSCs (ALDH−CD24−CD44+), and differentiated tumor cells (ALDH−non-CD24−CD44+). 

### 2.7. Functional Enrichment Analyses

For the functional enrichment analyses, we focused on the biological processes, molecular functions, and cellular components using the Panther classification system [42], while keeping the *p*-value of <0.05 for the number of genes enriched in each class. In addition, we analyzed the NeVOmics functional annotation program [43] with default parameters (FDR% 1.0). The minimum enrichment of 2 genes was used as the threshold cut-off. 

### 2.8. Genomic Analysis and Regulatory Mechanism of CanCord34 Genes

The whole chromosome amplification was studied using Progenetix [44]. The region of interest was aligned with the RefSeq gene track using the human genome assembly GRCh38.p13 and NCBI genome data viewer [45]. This allowed us to evaluate the status of the regulatory elements of the CanCord34 genes, such as the histone marks, transcription factors, enhancers, and other regulatory elements. We identified the motifs for specific transcription factors (TFs) in the CanCord34 genes within 1 kb of TSS by manually curating them from the ChiPBase v2.0 database [46]. We performed a cluster analysis to identify the shared TFs among the CanCord34 genes and represented them in a heatmap cluster matrix. Likewise, the gene enrichment analysis of the 34 genes was carried out using the ChEA gene set library and implemented using the MCF-7 cells [47,48]. The histone marks were classified into activating (green) and repressing (red) functions. A cluster matrix was employed to show the histone marks which have shared interactions between the CanCord34 genes. We used 15 as a cut-off, i.e., a histone mark shared by more than 15 genes. The CanCord34 genes were mapped against the reference genome hg19 using the IGV program [47]. Using the UCSC genome browser, we visualized the CanCord34 genes for all possible regulatory elements in different cell lines (https://genome.ucsc.edu/ accessed on 6 February 2019). A better representation of the active histone marks of the CanCord34 genes was achieved using a Circos plot [49]. 

### 2.9. Phenotypic Characterization Studies

We used the Genome RNAi database [50] to build the broad aspect of the gene’s function by annotating the gene functionally. To assess the role of the CanCord34 genes in cell viability, we used publicly available cell dependency datasets [51,52] and the recent approach used to analyze a given gene’s cellular fitness [53]. We downloaded and curated the data on genes for which fitness scores were available. The fitness score for the candidate gene was illustrated as a boxplot using ggplot() in R Bioconductor. 

### 2.10. Protein–Protein Interaction Analysis

To study the interactions between the CanCord34 genes, we employed the STRING program [54], an online protein–protein prediction tool. Each gene was represented as a node, and various interactions, such as gene fusion, evidence of neighborhood existence, protein co-occurrence, experimentally determined interactions, evidence of interactions curated from text mining, and evidence of interactions from the database, were represented as edges, and each type of interaction was represented in a different color.

### 2.11. Cell Culture and RNA Isolation

MCF-7, SKBR-3, MDA-MB-231, BT59, T47D, PC-3, OVCAR-3, OVCAR-8, AsPc-1, DU145, and BxPC-3 were maintained with RPMI 1640 (Invitrogen, CA). HT29, MiapaCa-2, and IMR32 were maintained with DMEM. U87MG was maintained in Eagle’s minimum essential medium. All the cells were supplemented with 10% FBS and 1X antibiotics/antimycotics in a humidified 5% CO_2_ at 37 °C. Total RNA was isolated from the cells using the TRIzol method according to the manufacturer’s protocol. DNA contamination from the isolated RNA was removed by DNase digestion using a Sigma DNase digestion kit. Briefly, the isolated RNA sample was mixed with a 0.1 volume of 10X DNase I buffer and 1µL of DNaseI followed by incubation at 37 °C for 20 min. The reaction was arrested by adding 2 µL of DNase inactivation reagent (stop solution) and by incubation at 70 °C for 10 min. A total of 2 µg of RNA was used for the cDNA synthesis using the SuperScript™ III Reverse Transcriptase kit (Invitrogen). 

### 2.12. Semi-Quantitative and Realtime PCR

Gene-specific primers obtained from Millipore Sigma (KiCqStart™ Primers ID numbers (H_EEF1D_1, H_TIGD5_1, H_PARP10_1, H_GPAA1_1, H_SCRIB_1, H_BOP1_1, H_PYCRL_1, H_GAPDH_1, H_C8ORF73_1, ACTB_1) were used for the amplification, and the PCR products were run on 1.5 % agarose gels and visualized using a gel documentation system (Bio-Rad). An SYBR green-based assay was performed to analyze the gene expression using specific primers. The primers were ordered from Millipore Sigma (KiCqStart™ Primers H_EEF1D_1, H_TIGD5_1, H_C8ORF73_1, H_BOP1_1, and H_ACTB_1). The gene expression was normalized using β-actin as an internal control. The fold change was calculated using the 2-ddCt method. A melt curve analysis was performed to test the specificity and quality of the qPCR amplicons. SYBR^®^ Premix Ex Taq™ II Tli RNase H Plus (Takara) was used for the qPCR analysis, performed in the StepOnePlus Real-Time PCR system (Applied Biosystem, CA, USA).

### 2.13. Analyses of Microvesicles from MSCs Exposed to Breast Cancer Cells

The co-culture used to select microvesicles/exosomes (MVs) from mesenchymal stem cells (MSCs) that were previously exposed to breast cancer cells and the utility of this system for studying the nature of MV-packed RNA cargo have been described previously [55,56]. As recently described, an equal number (5 × 10^4^) of MSCs (outer well) and MDA-MB231 cells (inner well) were co-cultured in a trans-well system for 24 h. After this, the inner well with the breast cancer cells and the media from the MSCs were removed, and the MSCs were washed and replaced with fresh exosome-free MSC media. After 48 h, the naïve and primed MVs were purified by ultracentrifugation and an exosome purification kit and analyzed using RNA sequencing data from the GSE138435 [56]. 

## 3. Results

### 3.1. Discovery of CanCord34 Genes in Breast Cancer

**Figure 1:**
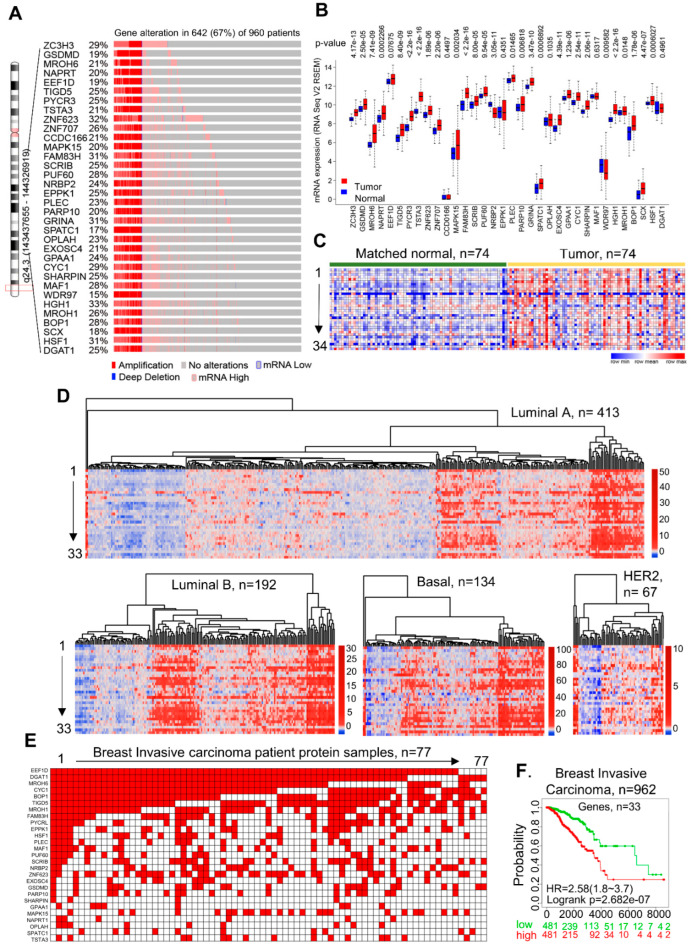
Overview of CanCord34 genes in breast cancer. (**A**) Dispersal of the genomic alterations in CanCord34 genes in breast cancer patients. Each row represents a gene, and each vertical line represents one patient sample. The alterations are defined with different colors and patterns. The data were taken from the cBioPortal platform. (**B**) Boxplot image showing differences between the expression patterns of CanCord34 genes in normal and matched tumor samples. The significance of the difference between the gene expression levels was evaluated using the Mann–Whitney–Wilcoxon test. (**C**) Expression of CanCord34 genes in normal and matched tumor samples. Except for CCDC166, all other genes were upregulated in the tumor samples compared to the normal samples. (**D**) Heatmap representation of the levels of the CanCord34 mRNAs using the heatmap algorithm of R for different subtypes. The samples with high expression, ~32% of the total samples, are clustered together. Many specimens belonging to the basal and luminal B (LumB) sub-types exhibited the upregulation of CanCord34 genes. (**E**) Heatmap representation of samples with genes upregulated at the protein level. Each row represents a gene, and the columns represent the tumor samples. Genes are sorted in descending order of gene expression in the tumor samples. (**F**) Kaplan–Meier survival plots of the CanCord34 genes. Out of 34 genes, 33 were taken to classify the samples, because one of the genes, the CCDC166, could not be mapped in the dataset. The survival plot for the CanCord34 genes was generated using the SurvExpress program.

During an investigation involving signaling-dependent intramolecular conformational changes in GSDMD, an established mediator of pyroptosis with an emerging role in breast and other epithelial cancer types [57,58], we observed that the levels of GSDMD expression are widely upregulated in breast cancer and that its amplification and expression are closely paralleled with an oncogene, the HSF1 [59] (Supplementary Figures S1 and S2), which was already studied in the Kumar laboratory in the context of PAK1-signaling-dependent phosphorylation in breast cancer. We recognized that both the GSDMD and HSF1 genes are 889.264 kb apart on the same locus on 8q.24 (Supplementary Figure S3) and 15,810.256 kb downstream from one of the most overexpressed oncogenes in human cancer, the MYC chr8 (127735434-127742951). Because of the substantial co-overexpression of GSDMD and HSF1 in breast cancer, this serendipity observation allowed us to postulate that the genes residing in between the GSDMD and HSF1 loci might also be upregulated in breast cancer. To test this hypothesis, we examined the expression of the genes residing in between the GSDMD and HSF1 in multiple breast cancer genomic datasets in the case of breast cancer (METABRIC) [28] and breast invasive carcinoma (TCGA, Firehose Legacy) (Supplementary Figure S4). Surprisingly, we found a widespread amplification and overexpression of the protein-coding genes between the GSDMD and HSF1 in breast cancer (Figure 1A,B, Supplementary Figures S3–S5). In addition, the genes flanking either orientation, the ZC3H3 and DGAT1, were upregulated in breast cancer. Notably, these protein-coding genes are continuously organized at a single locus, 8q24(143437655-144326919) (Supplementary Figure S3). Further, we found that the observed widespread mRNA overexpression of these genes could be detected with or without the amplification of the respective genes (Figure 1A,B, Supplementary Figure S4), suggesting that the expression of these genes, except for WDR97, might be regulated by factors beyond the CNV amplification, such as the transcription and post-transcription mechanisms of gene expression. Upon comparing the relative expressions of 34 genes in the matched normal and breast cancer tissue sets, we noticed a substantial co-overexpression of these genes in breast cancer, including TNBC, as compared to the matched normal tissue (Figure 1C, Supplementary Figure S6A), as well as the commonly used breast cancer cell lines (Supplementary Figure S6B), suggesting that these 34 genes might be preferentially overexpressed in breast cancer. The results of semi-quantitative RT-PCR assays confirmed the easily detectable overexpression and co-expression of a few selected examples of the CanCord34 genes of interest relative to the laboratory genes (i.e., TIGD5, EEF1D, PARP10, GPAA1, SCRIB, BOP1, PYCRL, and C8ORF73/MROH6) in the breast and other cancer cell lines (Supplementary Figure S7A,B). To obtain evidence regarding the effects of serum factors on the expression of the CanCord34 genes, we examined the effects of serum starvation for 72 h on the levels of EEF1D, TIGD5, C8ORF73/MROH6, and BOP1 in the breast cancer SKBR-3, cervical cancer SiHa, and neuroblastoma IMR-32 cell lines (Supplementary Figure S7C). We noted that the levels of EEF1D and C8ORF73 were increased upon serum starvation in all three cell lines, presumably due to the negative regulation of serum-derived factors, while the levels of TIGD5 and BOP1 were differentially affected by serum starvation. TIGD5 increased in SiHa, was reduced in IMR-32, and showed no substantial change in SKBR-3 cells, while we noted an increased and reduced BOP1 expression in the SKBR-3 and SiHa cells, respectively, and no substantial change in the IMR-32 cells. As serum contains both negative and positive growth factors, these preliminary observations suggested that the expression of the tested CanCord34 genes might be differentially affected in different cancer cell types.

The levels of expression of these genes in human tissues and a mixture of 16 normal human tissues were low (Supplementary Figure S8). The co-expression analysis of the 34 genes using the STRING tools showed a lack of co-expression of these genes in normal human tissues (Supplementary Figure S9). In subsequent studies, we referred to these 34 genes, from ZC3H3 to DGAT1, as “CanCord34” in regard to the co-overexpression of these genes, except for WDR97, which is amplified but not expressed.

The breakdown of breast cancer into four intrinsic sub-types indicated a relatively higher level of CanCord34 overexpression in luminal A and basal breast cancers compared to HER2 positive or luminal B breast cancers (Figure 1D) and a widespread co-occurrence of CanCord34 genes in triple-negative breast cancer (Supplementary Figure S10A–C). These observations suggested that the dysregulation of CanCord34 genes in breast cancer appears to be independent of the hormonal or HER2 status. A comparative overexpression analysis of the individual CanCord34 genes in breast cancer indicated that breast cancer generally shows a significant overexpression of most of the CanCord34 genes (1.5–14-fold, *p*-value less than 2.2 × 10^16^ to 0.6), except for a few genes. 

We further evaluated whether the expression of the CanCord34 genes correlates with the expression of the corresponding proteins using a detailed proteogenomic dataset of 77 breast cancer specimens [37]. We found that the CanCord34 gene expression correlates well with the protein expression. We believe that, in our case, the CanCord34 protein expression might be restricted in nature due to the smaller sample size of 77 and the technical details of the proteomic analysis performed by the investigators who deposited this dataset. However, we observed that the expression of the CanCord34 genes in breast cancer is correlated with the expression of most of the corresponding proteins in the same specimen and the levels of the CanCord34 mRNAs (Figure 1E and Figure S11). Prospective clinical and laboratory studies are also required to evaluate the status of the CanCord34 mRNA and proteins in newly collected breast tumor specimens (see Limitations and Outlook within the Discussion). To examine the clinical significance of CanCord34 overexpression in breast cancer patients, next, we performed a multivariate analysis of the CanCord34 genes using the Kaplan–Meier survival program, for which clinical data were available, except for data on CCDC166. We found that tumors with the overexpression of 33 of the CanCord34 genes correlated well with a significant reduction in the overall survival of the patients compared to breast cancer patients with low levels of these genes (Figure 1F).

Interestingly, when we performed the univariate Kaplan–Meier survival analysis of the CanCord34 genes in breast cancer, we noticed no significant overexpression of these genes individually, as compared to the multivariate analysis (Figure 1F) of 33 of the CanCord34 genes (Supplementary Figure S12). In these analyses, the high-risk groups with overexpressed CanCord34 genes could be separated from the low-risk groups with under-expressed genes immediately from the outset. Although several members of the CanCord34 genes have been reported to be sporadically upregulated in human cancer, the notion of continuously organized, co-overexpressed protein-coding CanCord34 genes at a single locus and notion that the overexpression of 33 of the CanCord34 genes is associated with poor survival among breast tumor patients were described here for the first time. Furthermore, 10 of the CanCord34 genes have not previously been reported to be overexpressed in breast cancer (see below). 

### 3.2. The Co-Overexpression of CanCord34 Family Members Is a Common Phenotype in Human Cancer

**Figure 2:**
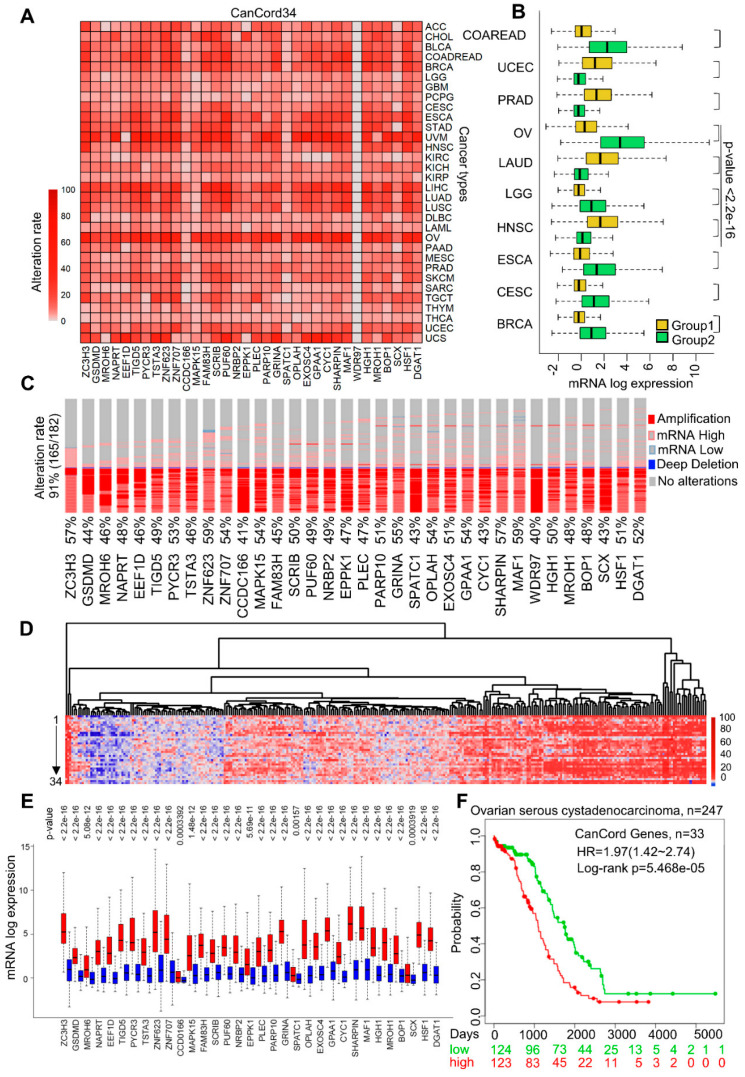
All-encompassing pattern of CanCord34. (**A**) A single-color heatmap represents the alteration rate of CanCord34 genes in 33 different cancer types. The increasing intensity of the color gradient is directly proportional to the increasing alteration rate. The X-axis represents the genes, and the Y-axis represents the TCGA abbreviation for the 33 cancer types. (**B**) Boxplot representation of the expression distribution of CanCord34 genes at the mRNA level for different cancers. Each cancer type was classified into two distinct groups based on CanCord34 expression. (**C**) Dispersal of alterations in the CanCord34 genes in ovarian cancer patients. Each row represents a gene, and each vertical line represents each patient sample. These genes are coordinately upregulated in patients at the mRNA level. The alterations are represented with different color codes and patterns. The data were taken from cBioPortal. (**D**) Heatmap representation of CanCord34 gene expression at the mRNA level, constructed using the heatmap algorithm of R for the ovarian cancer data. (**E**) Boxplot representation of the expression distribution between two groups identified using k-means clustering for each gene. The significance of the noticed difference between the gene expression levels was evaluated using the Mann–Whitney–Wilcoxon test. (**F**) Kaplan–Meier survival plot for the CanCord34 genes generated using the SurvExpress program.

The amplification of the chromosome 8q24 region, where the CanCord34 genes reside, is one of the most common genomic events in multiple cancer types [7,8,9,10,11]. We next determined whether the CanCord34 genes are co-overexpressed in other cancer types in addition to breast cancer. To this end, we first examined the expression of the CanCord34 genes with a 5% alteration rate of the total number of dysregulated genes in 32 cancer types. We noticed a widespread overexpression of CanCord34 genes in a substantial number of cancer types, ranging from 30% to 90% of the samples analyzed (Figure 2A, Supplementary Figure S13). The data in Figure 2B illustrate the levels of CanCord34 overexpression as compared to low-expressing specimens within the same cancer type for ten cancer types: TCGA breast invasive carcinoma (BRCA), TCGA cervical squamous cell carcinoma and endocervical adenocarcinoma (CESC), TCGA esophageal carcinoma (ESCA), TCGA head and neck squamous cell carcinoma (HNSC), TCGA brain lower-grade glioma (LGG), TCGA prostate adenocarcinoma (PRAD), TCGA lung adenocarcinoma (LUAD), TCGA uterine corpus endometrioid carcinoma (UCEC), and TCGA colon and rectum adenocarcinoma (COADREAD). For example, we found that 165 (91%) of 182 ovarian cancer specimens co-overexpressed CanCord34 genes (Figure 2C) and that this overexpression was independent of the pathologic staging of ovarian cancer (Figure 2D, Supplementary Figure S14). Consistent with these results, individual levels of the CanCord34 genes were substantially overexpressed in ovarian cancer compared to the low-expressing ovarian cancer specimens (Figure 2E). As expected from the observations in the preceding section, we noticed that the overexpression of 33 of the CanCord34 genes correlates well with a significant reduction in the overall survival of patients with ovarian cancer (Figure 2F). In brief, these findings suggest that the co-overexpression of CanCord34 genes may be a common event in multiple cancer types that is closely associated with the reduced survival of patients with overexpressed CanCord34 genes.

### 3.3. Towards an Understanding of the Functions of CanCord34 Genes

**Figure 3:**
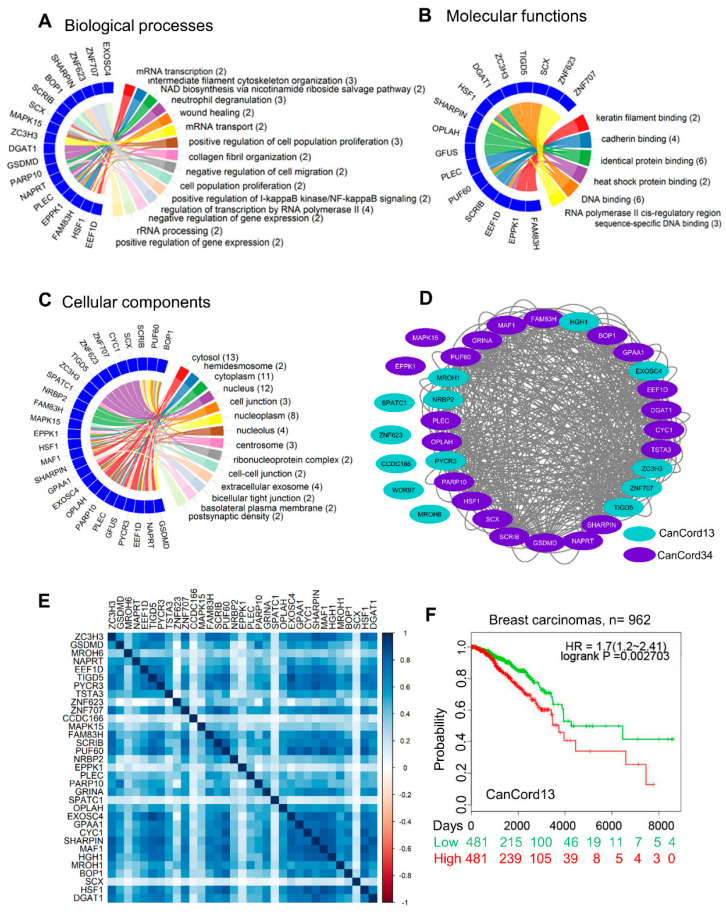
Functional enrichment analysis of CanCord34 genes. (**A**) Biological processes. (**B**) Molecular function. (**C**) Cellular components. Gene ontology enrichment analyses were performed using the NeVOmics functional annotation program and represented using Circos plots. (**D**) Circular network representing the CanCord34 inter-correlation. Node colors represent the class of genes as CanCord13 and CanCord34. (**E**) Functional protein–protein interaction using the online STRING platform. (**F**) Kaplan–Meier survival analysis of CanCord13 genes using the SurvExpress program.

To gain evidence regarding the potential functions of the CanCord34 genes, we utilized the widely used method of NeVOmics functional annotation [43]. The results of the biological process analyses indicated that the CanCord34 genes encode proteins with known or predicted diverse activities, such as transferase, hydrolase, ligase, and nucleic acid binding, transcription factors, and isomerase, lyase, and oxidoreductase activities (Figure 3A, Supplementary Figures S17 and S18). As cancer is a polygenic disease, the co-upregulation of CanCord34 genes with diverse encoded functions might contribute to the dysregulation of multiple biologic pathways with complementary functions during oncogenesis. The results of the molecular function analysis suggested that the majority of CanCord34 gene products could perform DNA and protein binding, as well as RNA polymerase binding activities (Figure 3B, Supplementary Figures S15 and S16). The subcellular localizations of the CanCord34-encoded proteins range from cell-to-cell junctions to the nucleus and to exosomes (Figure 3C), reinforcing the notion of the diverse functionality of the CanCord34 genes in multiple sub-cellular compartments of cancer cells. The regulation of immune processes, secretory activities, and extracellular microvesicles/exosomes emerged as the top functions of the CanCord34 genes (Supplementary Figures S15 and S16, Supplementary Table S1). Significant molecular functions were identified using *p*-values (Supplementary Table S1). To understand the nature of the co-expression, we used inter-correlation analysis and bioinformatics tools to visualize the experimentally proven and predicted interactions between the CanCord34 gene products in breast cancer (Figure 3D,E, Supplementary Figure S17B). In the case of breast cancer, we identified the overexpression of 32 of the CanCord34 transcripts with co-expression coefficients ranging from 0.002 to 0.89 (Figure 3E). We generally noticed a positive correlation in the various interactions between the CanCord34 genes (Figure 3E). For example, some highly interacting molecules include BOP1, EXOSC4, PUF60, MROH1, and TIGD5 (Supplementary Figure S17B). The number of nodes represents the total number of genes, and the 49 edges represent the experimentally proven and predicted interactions. On average, each node had a degree of 2.88, almost equal to the expected number of interactions, which was three. These results represent a high level of connectivity between the CanCord34 genes for a given function.

Similarly, we observed a high degree of overlap between the correlation study and STRING analysis, except for CCDC166 and SPATC1 (Supplementary Figure S17B, Figure 3D). Furthermore, CanCord34 genes such as MAPK15, EPPK1, ZNF623, WDR97, and MROH6 were shown to interact experimentally (Supplementary Figure S17A), as CanCord34 genes (red color nodes) share a high number of correlated genes (yellow color nodes), which suggests a high level of co-expression and shared functions (Supplementary Figure S17A). 

A closer examination of the biological functions of the CanCord34 genes suggested that, although several of the CanCord34 genes have known functions in the process of oncogenesis (such as HSF1, GSDMD, EEF1D, MAF1, etc.), we noticed that the role of 13 of CanCord34 genes remains poorly understood in breast cancer. For example, 10 of the CanCord34 genes (i.e., ZC3H3, MROH6, TIGD5, PYCR3, ZNF623, ZNF707, CCDC166, SPATC1, HGH1, and MROH1) have no publications in regard to breast cancer in PubMed, and 2 of the CanCord34 genes, namely NRBP2 and EXOSC4, had two studies and one study in breast cancer on PubMed as of 4 January 2022. Furthermore, three of the CanCord34 genes, namely MROH6, ZNF623, and CCDC166, have no prior PubMed publications in the context of cancer. The clinical relevance of this subset of 13 CanCord34 genes, operationally named CanCord13, was revealed by the observation that the overexpression of these genes correlates well with a significant reduction in the survival of breast cancer patients as compared to patients with low-CanCord13-expressing breast tumors (Figure 3F). These findings suggest that the co-overexpression of CanCord34 genes with diverse functions can impart distinctive cancer-relevant functions in breast cancer. 

### 3.4. CanCord34 Genes’ Regulation of Cell Viability

**Figure 4:**
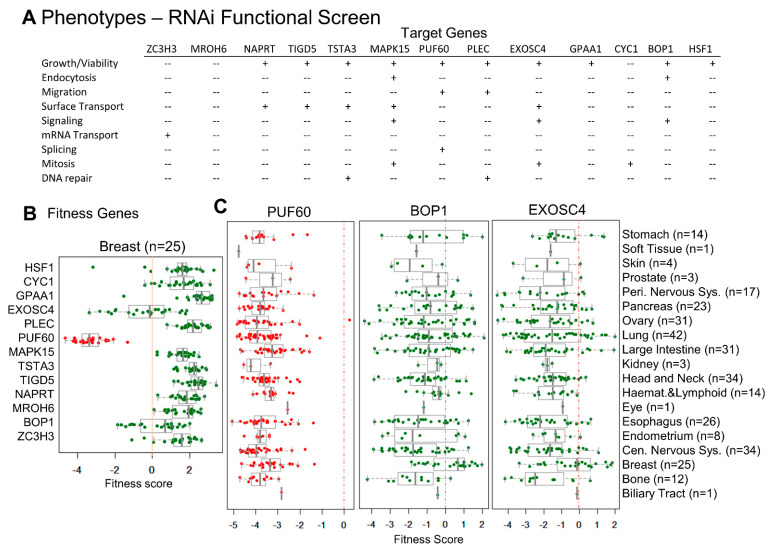
Towards the functions of CanCord34 genes. (**A**) Phenotypic analyses of a subset of CanCord34 genes using the functional RNAi screening database. (**B**) Distribution of a subset of CanCord34 genes as a cellular target with a significant fitness dependency effect upon its knockdown in the test breast cancer cell line. (**C**) Distribution of a subset of CanCord34 genes as a cellular target with a significant fitness dependency effect upon its knockdown in different cancer cell lines.

As CanCord34 genes encode proven or predicted proteins with diverse functions, we analyzed the nature of the phenotypes that might be evident upon selectively depleting these genes. In this context, we used a genome-wide RNAi functional screening dataset derived from physiologically relevant model systems [50]. We found that the knockdown of 13 of the CanCord34 genes correlated well with distinct quantifiable phenotypes (Figure 4A) and that the loss of the cell viability phenotype was one of the most frequently shared phenotypes with the knockdown of about 10 of these 13 genes of the CanCord34 family (i.e., EXOSC4, PUF60, BOP1, HSF1, GPAA1, PLEC, MAPK15, TSTA3, TIGD5, and NAPRT). The other commonly observed phenotypes of CanCord34 gene knockout included the cell surface transport (i.e., EXOSC4, TIGD5, MAPK15, TSTA3, and NAPRT), migration (i.e., PUF60 and PLEC), signaling (i.e., EXOSC4, BOP1, and MAPK15), splicing (i.e., PUF60), mRNA transport (i.e., ZC3H3), mitosis (i.e., EXOSC4, MAPK15, and CYC1), endocytosis (i.e., BOP1 and MAPK15), and DNA repair phenotypes (i.e., TSTA3 and PLEC). The STRING analysis showed the existence of experimental or predicted interactions between EXOSC4, PUF60, BOP1, HSF1, GPAA1, and CYC1 (Supplementary Figure S18). In brief, these findings provided evidence regarding the significance of several members of the CanCord34 genes in dysregulated cancer phenotypes. They strengthened the notion that the co-dysregulation of many cancer-relevant cancer processes might be driven by the co-upregulation of CanCord34 genes, at least in part. 

Next, we evaluated the impact of the knockdown of CanCord34 genes in supporting the cellular fitness phenotype of cancer cell lines. For this purpose, we used a cell dependency dataset representing a comprehensive CRISPR-Cas9 fitness screen, wherein 7470 genes were individually knocked down in 324 cancer cell lines from 30 different types of cancers [52]. The outcome for each gene is presented either as a fitness gene (i.e., knockdown of the test gene leads to the loss of cell viability) or a poor fitness gene for a given cell line [52,53]. We noticed that the cell dependency CRISPR-Cas9 screen dataset contained results pertaining to only 12 of the CanCord34 genes (Figure 4B). Among these 12 CanCord34 genes, the knockdown of PUF60, EXOSC4, or BOP1 was accompanied by the loss of cell viability in breast cancer cell lines (Figure 4B). Interestingly, the loss of PUF60, EXOSC4, or BOP1 was also closely correlated with the loss of cell viability in the cell lines of multiple cancer types, except for the skin, eye, and biliary tract cell lines (Figure 4C, Supplementary Figure S21), suggesting the involvement of EXOSC4, PUF60, and BOP1 in the viability of breast cancer cells, in addition to other cancer-relevant functions. 

The BOP1 has previously been shown to promote cancer progression phenotypes and the epithelial to mesenchymal transition in several cancer type cell lines [60,61,62,63]. Similarly, EXOSC4 (exosome component 4), a component of the RNA exosome complex [64], regulates growth, WNT signaling, and surface transport in cancer cells. EXOSC4 overexpression in liver cancer cells has been attributed to the hypomethylation of its promoter, and EXOSC4 downregulation inhibits the growth and invasiveness of liver cancer cells [65]. EXOSC4 overexpression is also considered as a growth-promoting gene event in ovarian cancer, as it stimulates WNT signaling, and it enhances the invasion and tumor-forming potential of colon cancer cells [66]. The other functional molecule of interest in cancer progression is PUF60 (poly(U) binding splicing factor 60), a component of the spliceosome [67]. PUF60 is widely overexpressed in bladder cancer cells. It supports viability and growth due to its ability to regulate Aurora A transcription [68].

In contrast, PUF60 overexpression in renal cancer cells stimulates telomerase reverse transcriptase (TERT) transcription due to PIF60’s binding to the TERT promoter [69]. PUF60 also contributes to breast cancer progression via the PUF60-dependent downregulation of PTEN, stimulating the PI3-kinase pathway [70]. In brief, these results exemplify the concept that the diverse components of CanCord34 genes play important roles in supporting the cellular viability and fitness of multiple cancer cell types.

### 3.5. Upregulation of CanCord34 Family Members in Cancer Stem Cells

Among the molecules of interest discussed in the preceding paragraph, BOP1 and EXOSC4 have previously been linked to stem cell biology and were shown to play roles in cell viability and fitness (this study). For example, BOP1 overexpression confers chemoresistance on TNBC cells via stimulating the WNT signaling and cancer stem cell phenotypes, such as aldehyde dehydrogenase 1A1 (ALDH1A1) [60]. However, there is also evidence to suggest that BOP1 is a downstream target of the WNT/beta-catenin pathway in colorectal cancer cells [71], suggesting that additional studies are required to further understand the role of BOP1 in cancer stem cells. Secretory extracellular vesicles and/or exosomes and EXOSCs also play an essential role in sustaining the progenitor activity of the stem cell population during self-renewal in the epidermis [72] and fine-tuning the balance between growth and maturation during erythropoiesis [73]. There is also evidence to implicate the involvement of extracellular secretory vesicles in maintaining redox homeostasis in pluripotent stem cells [74]. As regulators of growth, surface transport, exosomes, and signaling pathways have been linked in cancer stem cell biology [75], next, we explored the role of the CanCord34 genes in the context of human stem cells. 

**Figure 5:**
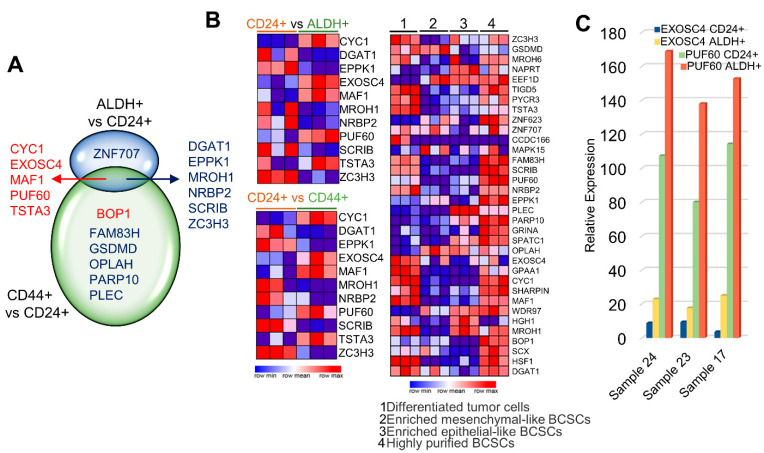
CanCord34 genes in cancer stem cells. (**A**) Venn diagram representing the CanCord34 genes reported to be involved in the stem cells. Upregulated genes are represented in red, whereas downregulated genes are in blue. (**B**) Heatmap representation of the CanCord34 gene expression in ALDH+ vs. CD24+ cell clusters (top left), CD24+ vs. CD44+ cells, and differentiated tumor cells, enriched mesenchymal-like BCSCs, enriched epithelial-like BCSCs, and highly purified BCSCs. The image was constructed using the heatmap package of Gene Patterns. (**C**) Bar chart representing the expressions of EXOSC4 and PUF60 in the sample sets of ALDH+ and CD24+ cells.

Stem cells with ALDH+ and CD24+/CD44+ markers exhibit phenotypes of epithelial-like and mesenchymal-like mammary cells [76]. Next, we assessed the levels of CanCord34 genes in ALDH+ or CD24+/CD44+ stem cells in a single-cell RNA sequencing dataset derived from human mammary cells isolated from normal breast cells [40]. We found that five (i.e., CYC1, EXOSC4, MAF1, PUF60, TSTA3, and BOP1) of the CanCord34 genes are upregulated in and shared by the ALDH+ and CD24+/44+ mammary stem cells, and that upregulated BOP1 is largely expressed in the CD44+ stem cells (Figure 5A,B left panel). To better understand the involvement of these CanCord34 genes in stem cells, we also curated another stem cell dataset. In general, we found a higher expression of CanCord34 genes in the differentiated tumor cells, except for CCDC166, GSDMD, TSTA3, and highly purified BCSCs cells, compared to the other stem cell types (Figure 5B right panel). All three genes (i.e., PUF60, EXOSC4, and BOP1) with a suspected role in viability and stem cell biology were also detectable in the CD24+ mesenchymal-like and ALDH+ epithelial-like cells. However, the levels of expression of these genes were higher in the ALDH+ mammary cells (Figure 5C). We also observed that the levels of several CanCord34 genes, such as MAFA, NAPRT, EEF1D, ZNF623, MAPK15, EPPK1, PLEC, PARP10, GRINA, SPATC1, OPLAH, and WDR97, were higher in the tumor cells as compared to their expression in the purified BCSCs (Figure 5B). This suggests the potential role of certain members of the CanCord34 family in the differentiation of tumor cells, and that the dysregulated expression of such CanCord34 genes could be involved in maintaining the stemness of cells in the case of differentiated tumor cells.

**Figure 6:**
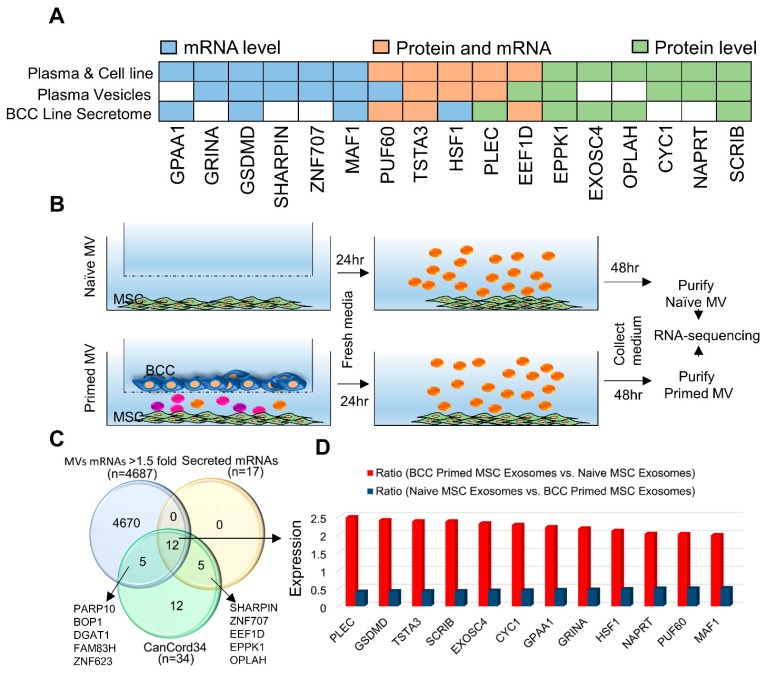
CanCord34 in secretory functions. (**A**) The literature curation of CanCord34 genes shows a subset of CanCord34 genes as secreted mRNA, proteins, or both. (**B**) Cartoon showing the model system used. (**C**) Venn diagram showing the overlap of the 12 upregulated mRNAs present in MSC-derived extracellular vesicles (*n* = 4686, >1.5-fold with a *p*-value 0.05), secreted CanCord34 mRNAs and/or proteins identified in public databases (*n* = 17), and CanCord34 genes (*n* = 34). (**D**) Bar chart showing the upregulation of 12 secreted CanCord34 genes in MSCs incubated with primed exosomes vs. naïve MSC exosomes.

Our observation that three diverse members of the CanCord34 genes, namely PUF60, a component of spliceosome, EXOSC4, a component of exosome complex with exoribonuclease, and BOP1, a ribosomal biogenesis factor, exhibit shared roles in stem cell phenotypes and cell viability raised the possibility of functional interactions between these genes. These observations suggest that members of CanCord34 genes are likely to contribute to the course of cancer progression due to their role in supporting the cancer stemness phenotypes. In support of this notion, we found that these genes were induced in the breast cancer cell–MSC culture system and the component of secreted MVs from mesenchymal stem cells (see Figure 6 below). 

### 3.6. CanCord34 Family Members as Components of Extracellular Microvesicles and Vesicles/Exosomes

The results from the preceding sections suggest that members of the CanCord34 gene family might be involved in secretory functions and extracellular vesicle biology. To explore this notion, we determined whether CanCord34 genes are components of secreted extracellular vesicles or exosomes using a publicly available secretome database [39]. The results indicated that 17 of the CanCord34 genes’ transcripts, proteins, or both could be detected in the secretomes and plasma vesicles, including the EXOSC4 and PUF60 proteins, in the breast cancer secretome datasets (Figure 6A). These observations reinforce the notion that several members of the CanCord34 family are components of the extracellular vesicles and might be involved in cellular communications. It is generally accepted that extracellular vesicles play inherent roles in cellular communication and in reprograming the transcriptomes of the recipient cells and conferring therapeutic resistance in breast and other cancer types [77,78,79], presumably due to the molecular actions of biomolecules within the secreted vesicles during the process of cell–cell communications. There are also examples of the role of secreted vesicles in transferring the effect of breast cancer cells upon the polarization of the macrophages and, in turn, contributing to the metastasis of the lymph nodes and breast cancer tumor dormancy [55,56,79]. 

Next, we validated the presence of the CanCord34 genes in the secreted MVs. To this end, we took advantage of a widely used, physiologically relevant co-culture system at the interface of extracellular vesicle and stem cell biology developed in the laboratory of one of the co-authors (i.e., P.R.) [55,56]. In this model, the nature of the MVs secreted by MSCs previously exposed to breast cancer cells showed the distinct nature of the mRNA cargo compared to MSCs that were never exposed to cancer cells (Figure 6B). These MVs can induce breast cancer dormancy in the bone microenvironment [55,56]. The results of the RNA sequencing data (GSE138435 and reference, Sandiford et al., 2021) of the purified MVs identified increased levels of 12 of the 17 secreted CanCord34 genes (including the EXOSC4 and PUF60 mRNAs) in the primed MVs secreted by MSCs which were exposed to breast cancer cells (Figure 6C,D). The levels of secreted CanCord34 genes in the primed MVs ranged from 1.59-fold to 2.48-fold their levels in the naïve MVs. Interestingly, one of the 14 secreted CanCord34 genes, ZNF707, has not previously been studied in breast cancer. As ZNF707 has been predicted to bind to nucleic acid, it might potentially be involved in gene expression during cellular communications involving the MSCs. To understand the clinical significance of the 12 secreted CanCord34 genes in breast cancer, which are also co-overexpressed, we analyzed the impact of the overexpression of these 12 genes on the overall survival of breast cancer patients. We found that an increased expression of 10 out of these 12 genes (i.e., PLEC, GSDMD, SCRIB, EXOSC4, CYC1, GPAA1, GRINA, HSF1, PUF60, and MAF1, while TSTA3 and NAPRT could not be mapped to the dataset) was correlated with poor overall survival breast cancer patients (Supplementary Figure S19).

Our finding that a subset of CanCord34 genes are components of the MVs secreted by MSCs is significant, because the primed MVs are required for the final transition step through which breast cancer cells dedifferentiate into dormant chemo-resistant cancer stem cells [55,56]. In this context, recently, MSC-derived MV cargo has been proposed to promote dedifferentiation via the WNT pathway [56], which has also been shown to be involved in the actions of BOP1 and EXOSC4 in stem cells [60,71,72]. Collectively, these findings support the notion of the role of members of the CanCord34 genes in the cellular communications between the breast cancer cells and MSCs for the conferral of chemoresistance. These studies suggest that the expression of certain members of the CanCord34 family might be modified during cellular communications, and that 12 of the CanCord34 genes are also components of the extracellular vesicles. These results raise several interesting scientific avenues for follow-up to assess the impacts of the MSCs on the biology and genomics of breast cancer cells, and vice versa, within the context of EXOSC4 and PUF60, as well as other CanCord34 genes secreted in the MVs, in regard to the progression and dormancy of breast cancer. 

### 3.7. Search for Shared Mechanisms of the Co-Upregulation of CanCord34 Genes

Although several members of the CanCord34 gene family have been reported to be sporadically upregulated in cancer, the notion of the co-overexpression of continuously organized genes at a single locus with diverse functions in breast cancer has not been described before. Our present results provide evidence of the widespread co-overexpression of CanCord34 mRNAs in breast and other human cancers. For example, we identified the co-overexpression of 30 or more transcripts of the CanCord34 genes in 18 human cancer types (Supplementary Figure S13) and the overexpression of 32 CanCord34 transcripts with co-expression coefficients ranging from 0.002 to 0.89 in breast cancer (Figure 3E). Our observations of the high co-overexpression frequency of CanCord34 mRNAs in human cancer and the overexpression of CanCord34 genes in the absence of genomic amplification raised the possibility of shared mechanisms of co-overexpression among the CanCord34 genes in breast cancer. Examples of such shared mechanisms of transcriptional stimulation may include, but are not limited to, the presence of common interacting motifs/responsive elements in the CanCord34 gene promoters for transcription factors and the engagement of shared activating histone marks for the recruitment of distinct components of the chromatin remodeling machinery, etc. [80,81].

**Figure 7:**
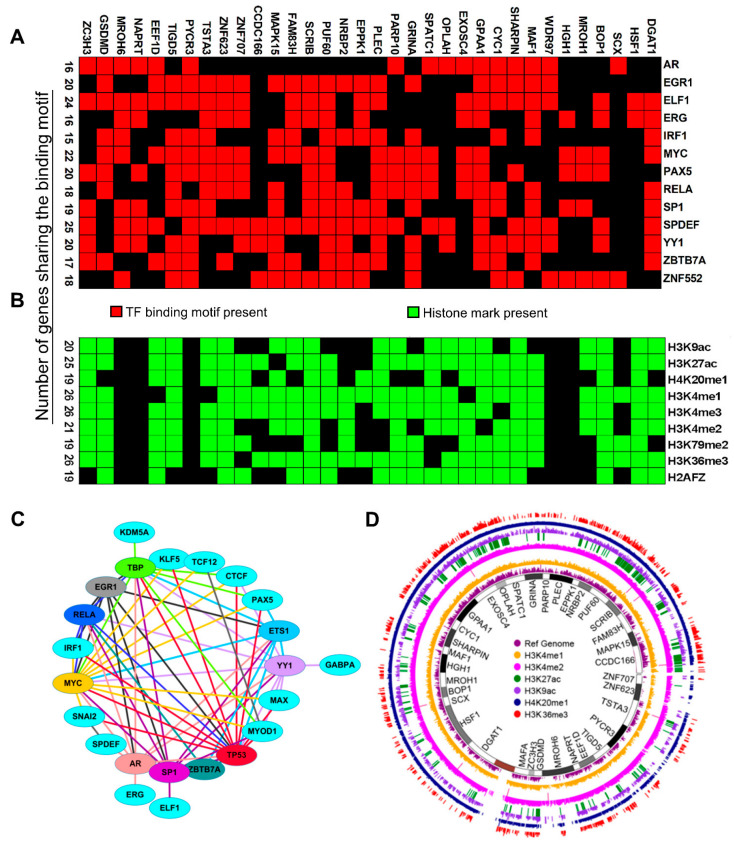
Shared regulatory elements of CanCord34 genes. (**A**) Shared transcription factors associated with the CanCord34 genes within 1 kb from the TSS. (**B**) A cluster matrix showed the activating histone marks shared by the CanCord34 genes in MCF-7 cells. (**C**) Interactions and heterodimerization of TFs with the base TFs (in which interacting response elements are present) in CanCord34 genes. (**D**) Histone mark enrichment analysis of CanCord34 genes and the status of activated histone marks, represented as a Circos plot.

To evaluate these postulations, next, we examined the promoters of the CanCord34 genes for the presence of established interacting motifs for DNA-interacting factors within 1 kb upstream and downstream of the transcription initiation sites (TSS). As expected, we identified the presence of multiple motifs for DNA-interacting factors that were shared by the CanCord34 genes (Supplementary Figures S20 and S21). For example, the SPDEF, CTCF, ELF1, and MYC motifs can be found in the promoters of 26, 24, 24, and 23 genes of the CanCord34 family, respectively. In contrast, the promoters of the GPAA1, MAPK15, SPATC1, and MAF1 genes contain interacting motifs for many shared DNA-interacting factors. Representative data pertaining to the commonly identified transcription-factor (TF)-binding motifs in the CanCord34 genes are shown in Figure 7A. The binding of nuclear receptors to the target DNA is regulated by multiple mechanisms [82,83]. The formation of homo- and hetero-dimers among TFs is essential for regulating the gene expression [84,85,86]. Our observation that 13 out of the 23 TFs of interest are known to interact and heterodimerize with other TFs (Figure 7C) is important, as the formation of homo- and hetero-dimers among TFs is an essential step in the regulation of gene expression [82]. Many DNA-interacting proteins are known to be phosphoproteins and become activated upon phosphorylation by signaling kinases. In this context, it is interesting to note that many of the 23 TFs of interest are, indeed, phosphoproteins by nature [86], suggesting that the functionality of such TFs is expected to be positively influenced by hyperactivated signaling kinases in cancer cells and, in turn, might contribute to the observed overexpression of the CanCord34 family members. Interestingly, because one of the co-overexpressed CanCord34 genes is MAPK15 (also referred to as ERK7 or ERK8), a commonly hyperactivated signaling kinase in breast and other human cancers [87,88], we postulate that MAPK15 overexpression can potentially create a positive loop between the persistent MAPK15 activation, the phosphorylation of its predicted nuclear substrates, and the upregulation of the target CanCord34 genes. 

In addition to TF- and/or DNA-interacting factors, the process of gene stimulation is profoundly influenced by the engagement of chromatin remodeling complexes with the target genes via activated histone marks (Figure 7B). Hence, we determined the recruitment of these activated histone marks to the CanCord34 genes using the UCSC genome browser. We identified the combinatorial recruitment of all nine activated histone marks to 19 to 27 of the CanCord34 family genes (Figure 7B,D, Supplementary Figure S21). For example, the H3K4me1, H3K4me2, and H3K36me3 marks are recruited to 27 CanCord34 genes, including EXOSC4, PUF60, BOP1, and MAPK15. A comprehensive atlas of the activating and repressive histone marks is illustrated in Figure 6D and Supplementary Figure S22, respectively. A closer examination of the promoters of a few candidate CanCord34 genes suggested that some of the noticed activated histone marks are in the vicinity of the TF-interacting motifs within the promoters of EXOSC4, PUF60, BOP1, CYC1, or TIGD5 (Supplementary Figure S23), suggesting that activating histone marks (and interacting chromatin remodeling complexes) and DNA-interacting factors might cooperate during the process of gene stimulation. In addition to these mechanisms, other underlying shared candidate mechanisms of the co-overexpression of CanCord34 genes include shared enhancer and transposons elements, long-range chromatin looping, etc. (Supplementary Figure S24). 

## 4. Discussion

The results presented here have describe the overexpression of continuously organized protein-coding genes in breast and other cancer types. As CanCord34 genes reside at 8q24.3, which is known to be one of the highly amplified chromosomal regions in breast cancer, our findings re-emphasize the significance of coordinately co-overexpressed genes in breast and other cancers. Although several members of the CanCord34 genes have been reported to be sporadically upregulated in human cancer and the fact that 11 (i.e., SHARPIN, HSF1, BOP1, HGH1, EXOSC4, ZC3H3, DGAT1, MAF1, PUF60, PYCR3, and GPAA1) of the CanCord34 genes are part of the pan-cancer SARPIN signature of 20 genes [6], the notion of continuously organized, co-overexpressed protein-coding CanCord34 genes at a single locus was described here for the first time. The results presented here also revealed ten new overexpressed genes with no prior PubMed publications (i.e., ZC3H3, MROH6, TIGD5, PYCRL3, ZNF623, ZNF707, CCDC166, HGH1, SPATC1, and MROH1) in regard to breast cancer. Our multivariant Kaplan–Meier survival curves established the clinical relevance of the CanCord34 genes or their subsets in breast cancer, showing highly significant associations between the overexpression of CanCord34 genes and the reduction in the disease-free survival of breast cancer patients. As the members of the CanCord34 genes belong to proteins with diverse biological activities and functions, this suggests that the persistent co-overexpression of cancer-relevant CanCord34 genes is likely to contribute to a set of complementary functions which might be common across multiple cancer types. These conclusions are consistent with the notion that cancer is a polygenic disease at large and involves the co-dysregulation of several functional pathways and underlying regulatory molecules. For example, the present study provided new insights into the involvement of several members of the CanCord34 genes (i.e., EXOSC4, PUF60, and BOP1) in cell viability, cancer stem cell biology, and extracellular communications via secreted plasma vesicles. Our findings provide initial evidence regarding the existence of shared mechanisms of the transcriptional co-upregulation of multiple members of the CanCord34 genes involving the presence of the cassettes of common TF-binding motifs and modified histone marks shared by the CanCord34 genes and the potential utilization of these molecules in a combinational manner, which might account for the noticed high frequency of co-upregulated CanCord34 genes. 

Breast cancer, being a heterogenous disease, often involves the dysregulation of multiple pathways. Though some cancers are driven by mutant genes and/or addicted to certain pathways, the pathobiology of most cancer types, including breast cancer, is polygenic. At the molecular level, the dysregulation of regulatory pathways in cancer cells is believed to be driven by the co-overexpression of genes. To better understand breast cancer’s pathobiology and unearth new facets of the shared dysregulation of molecular pathways, one potential approach is to search for coregulated genes with roles in diverse functions beyond one cancer type. We addressed this conundrum in the present discovery study through a serendipity observation. Here, for the first time, we described the notion of the coordinated co-dysregulation of a set of 34 continuously organized protein-coding CanCord34 genes at a single locus and demonstrated that the overexpression of 33 of the CanCord34 genes is associated with poor survival among breast tumor patients. Because of the co-dysregulation of multiple genes with roles in diverse cancerous processes, our findings suggest that the persistent co-upregulation of CanCord34 genes might promote the dysregulation of complementary functions in the breast (and other cancers) and, in-turn, strengthen the concept that breast cancer, being polygenic, may best be addressed through polygenic approaches.

Limitations and Outlook: As in the case of other new findings, the results presented here open up new research avenues in cancer biology. Examples of these new questions include, but are not limited to, the further experimental validation of the postulations made here, the definition of the regulatory mechanisms of gene expression of the numerous newly described co-upregulated molecules, the revelation of the inner workings of the molecular basis of the coordinated co-upregulation of the CanCord34 transcripts, the search for the upstream modifiers and downstream effectors of specific CanCord34 genes/proteins, and efforts to further understand the prognostic and therapeutic value of CanCord34 genes, etc. In addition, these studies may pave the way for the design of a functional genomic screen in an appropriate whole animal model system wherein the CanCord34 genes could be symmetrically depleted in a combinatorial manner, followed by knocking in the candidate genes to restore specific functions. Such studies are planned for the next phase of this research. Prospective clinical and laboratory studies are also required to evaluate the status of the CanCord34 mRNA and proteins using a set of newly collected breast tumor specimens. Future studies are needed to begin to overcome many of these current limitations, i.e., the functional validation of the CanCord34 genes for specific phenotypes, such as those related to cell viability, DNA damage response, aggressiveness, therapeutic resistance, etc., using gain- and loss-of-function screening studies of genes of interest using a combinatorial-based experimental strategy with appropriate internal controls, including the modulation of the status of the expression of one or two genes alone, in appropriate model systems. The present study strengthens the idea that cancer, being a polygenic disease, may best be addressed through polygenic approaches and solutions.

## Data Availability

Not applicable.

## Supplementary Materials

The following supporting information can be downloaded at: https://www.mdpi.com/article/10.3390/cells11233806/s1, Figure S1: Similar patterns of alterations in GSDMD and HSF1; Figure S2: Overexpression of GSDMD and HSF1 in human cancer; Figure S3: Chromosomal status and genomic region; Figure S4: Widespread upregulation of CanCord34 genes in breast cancer; Figure S5: Correlation between the mRNAs and CNVs of CanCord34 genes; Figure S6: Status of CanCord34 in the matched normal and breast cancer cells in 51 breast cancer cell lines; Figure S7: Expression of some CanCord34 gene family members in cancer cell lines; Figure S8: CanCord34 expression in different clusters of normal tissues and mixtures of tissues; Figure S9: Status of the co-expression of CanCord34 genes in human tissues; Figure S10: Status of the co-expression of CanCord34 genes in human tissues; Figure S11: Proteogenomic expression of CanCord34 genes in breast cancer dataset; Figure S12: The Kaplan–Meier survival plots of individual CanCord34 genes in breast cancer; Figure S13: Overview of alterations/upregulation of CanCord34 genes in human cancer with a minimum alteration rate of 5%; Figure S14: Disease-stage-wise representation of CanCord34 gene expression in ovarian cancer; Figure S15: Gene ontology enrichment analysis of CanCord34 genes; Figure S16: CanCord34 genes as secretory molecules using the data retrieved from the VESICLEPEDIA; Figure S17: Interconnections between CanCord genes in breast cancer; Figure S18: Curated functions from the Genome RNAi database and evidence of protein–protein interactions from STRING analysis of six of the CanCord34 genes which have shown potential roles in the cell viability; Figure S19: The Kaplan–Meier survival plots of 10 secreted genes in breast cancer; Figure S20: The transcription factor motifs present in the Cancord34 genes within 1 kb up- and down-stream of TSS; Figure S21: Transcription-factor-binding motifs in CanCord34 genes and their heterodimerization with other TFs; Figure S22: Circos plot showing various activated and repressive histone marks shared by the CanCord34 genes; Figure S23: Examples of TFs and histone marks shared by the genomic coordinates of a few members of the CanCord34 genes using the UCSC genome browser; Figure S24: The genomic coordinates of CanCord34 genes. The genomic coordinates and possible regulatory elements were visualized using the UCSC genome browser in different cell lines; and Table S1: coregulation map of CanCord34 in the human proteome [37,44,51].
