# Supplementary material for: The Revelation of Continuously Organized, Co-Overexpressed Protein-Coding Genes with Roles in Cellular Communications in Breast Cancer
Source: Cells. 2022 Nov 28;11(23):3806. doi: 10.3390/cells11233806 (PMC9741223; doi:10.3390/cells11233806)
Supplement: Supplementary file 1 [file cells-11-03806-s001.zip › cells-1955049-supplementary.pdf]

The Revelation of Continuously Organized, Co Overexpressed Protein-Coding Genes with Roles in Cellular Communications in Breast Cancer

Aswathy Mary Paul, Revikumar Amjesh, Bijesh George, Deivendran Sankaran, Oleta A. Sandiford, Pranela Rameshwar, Madhavan Radhakrishna Pillai and Rakesh Kumar

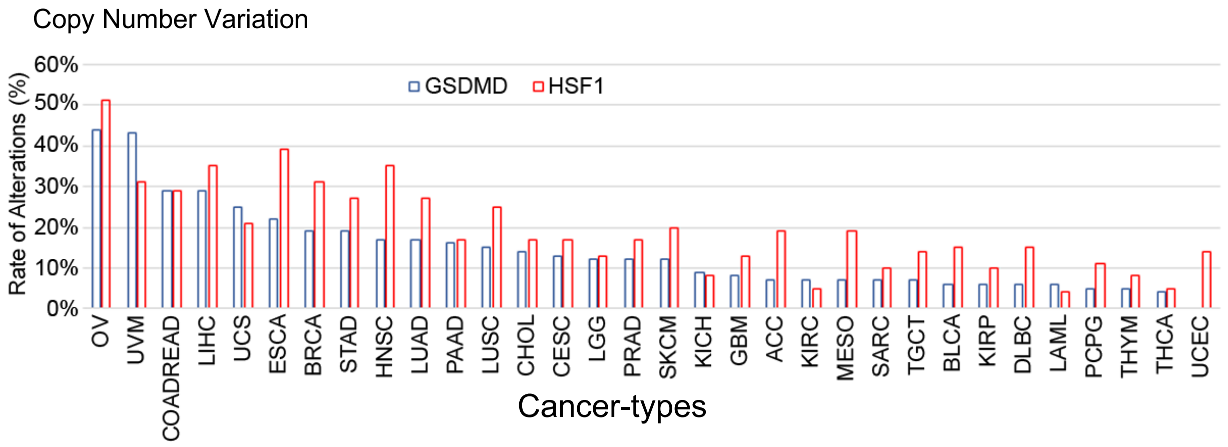

Supplementary Figure S1: Similar pattern of alterations of GSDMD and HSF1. Percentage of the CNV alterations of GSDMD and HSF1 across the human cancer. The X-axis represents cancer types described as per the TCGA abbreviation, and Y-axis represents the rate of alterations. Data shown here is based on GSDMD alteration rate in the cBioportal database.

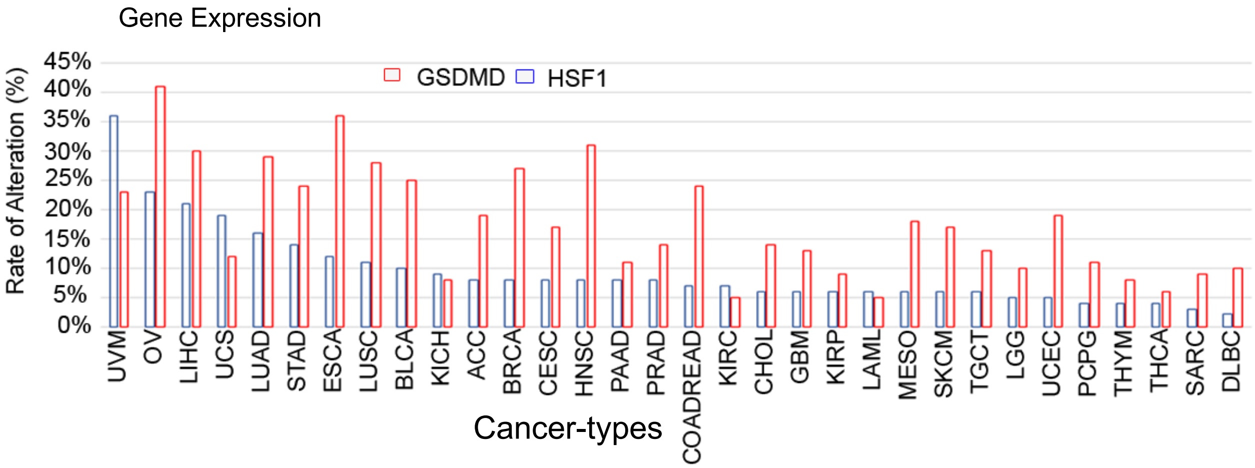

Supplementary Figure S2: Overexpression of GSDMD and HSF1 in human cancer. Percentage of alterations of GSDMD and HSF1 across different cancer at the mRNA level. The X-axis represents cancer types described as per the TCGA abbreviation, and Y-axis represents the rate of alterations. Data shown here is based on GSDMD alteration rate in the cBioportal database.

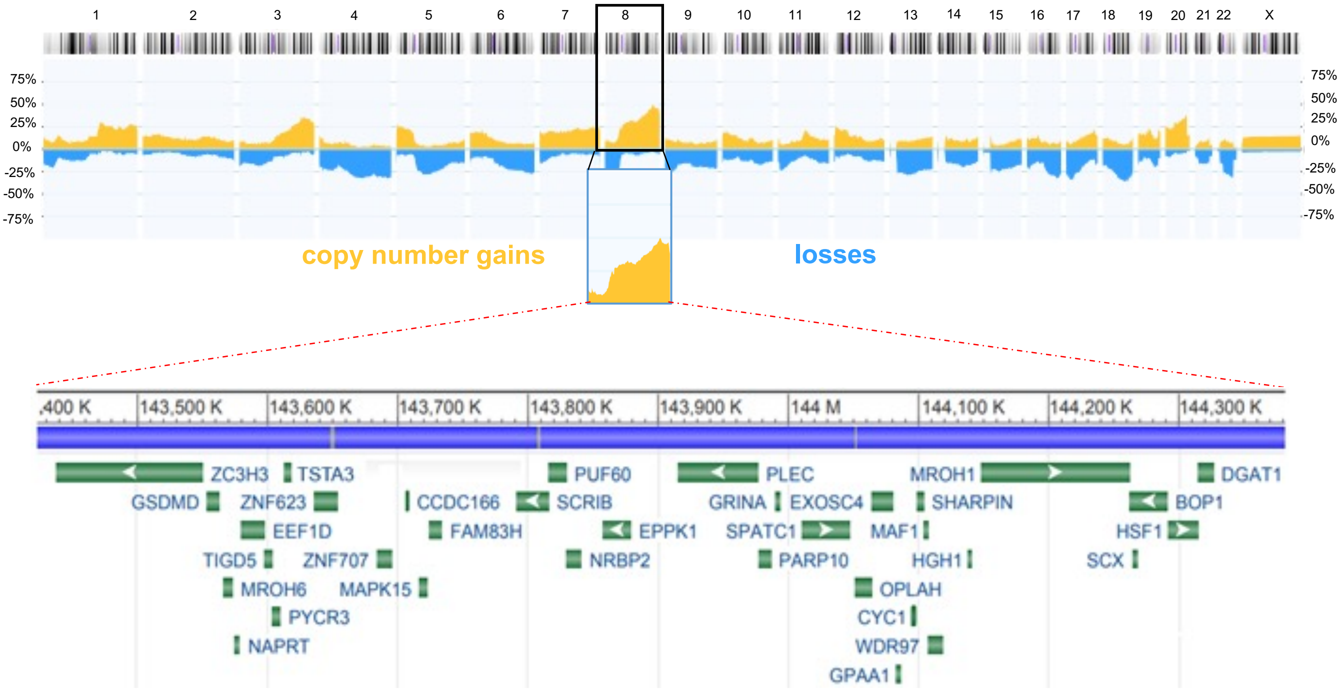

Supplementary Figure S3: Chromosomal status and genomic region. Progenetix analysis (44) of the amplified chromosome 8 in breast cancer. CanCord34 genes reside on 8p24, 143437655 – 144326919 region.

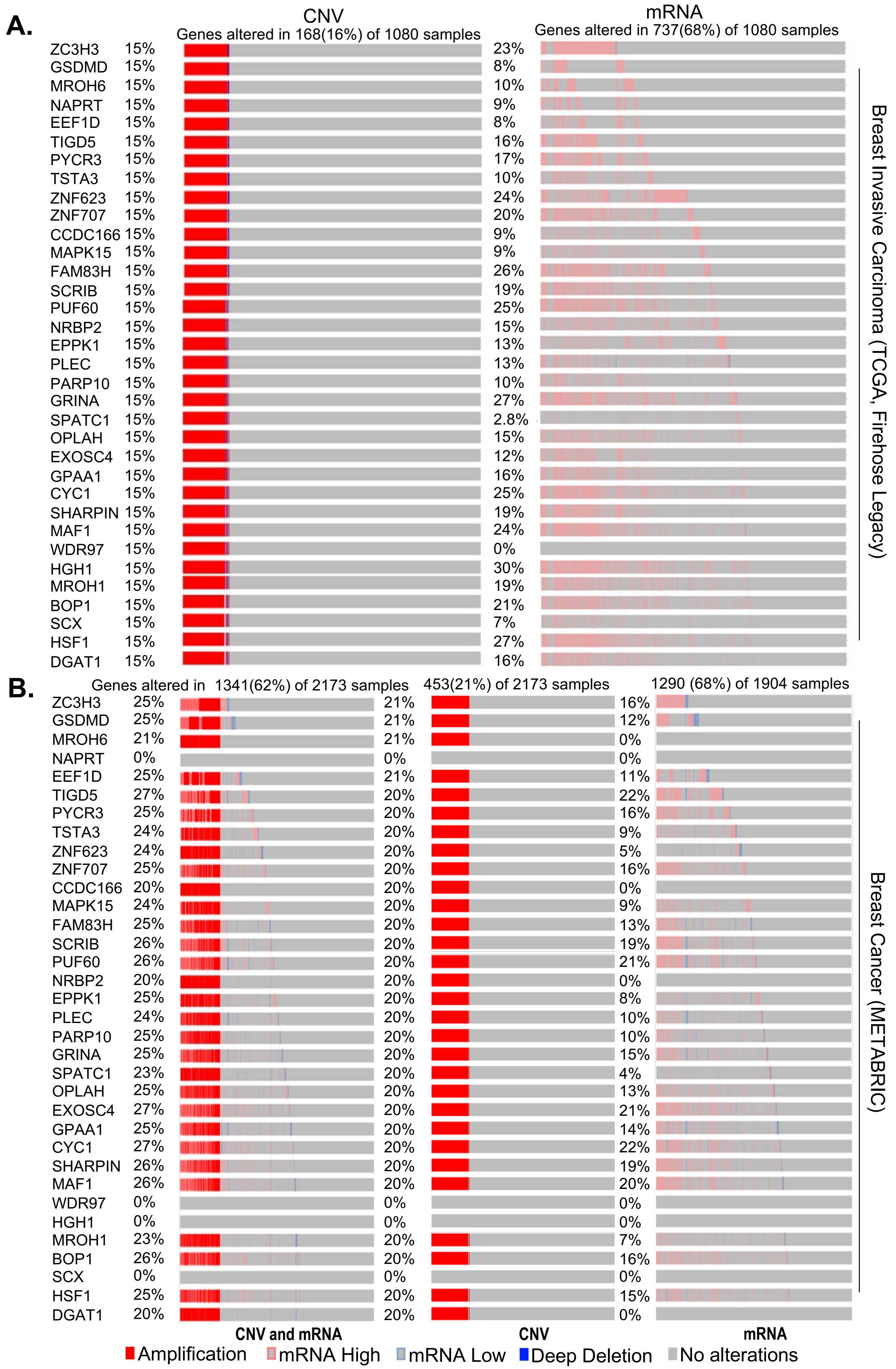

Supplementary Figure S4 Conti.

C.

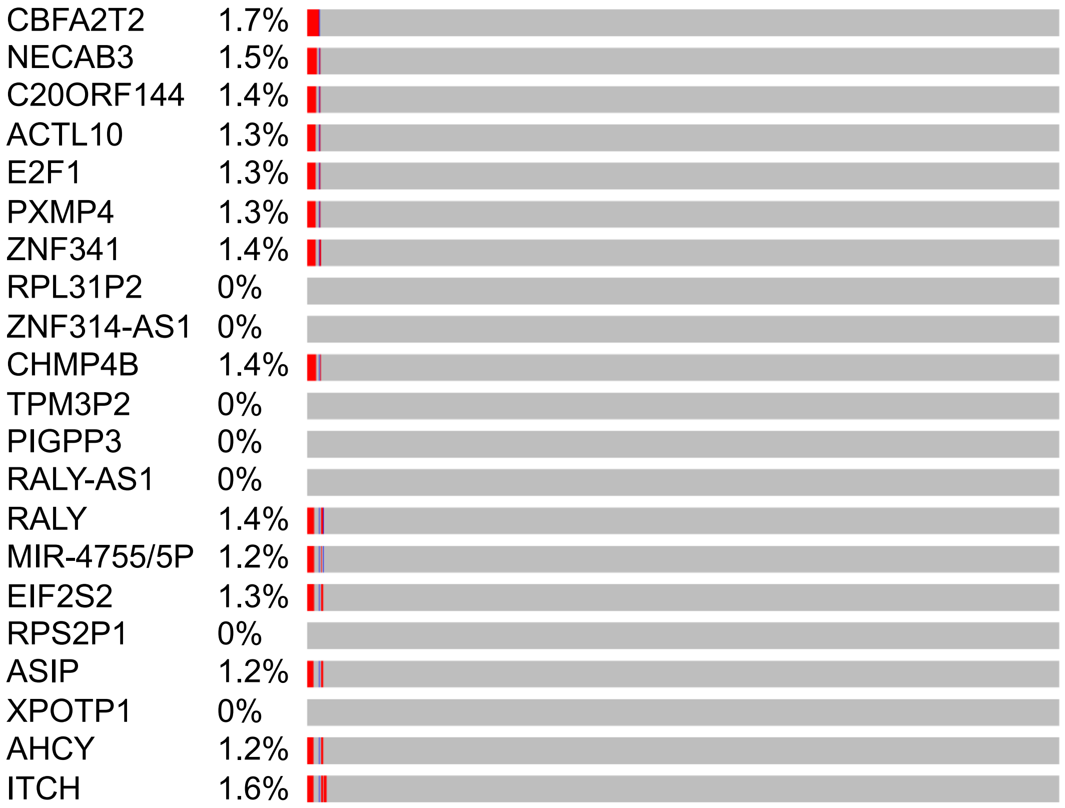

Supplementary Figure S4: Widespread upregulation of CanCord34 genes in breast cancer. **(A)** Breast Cancer data from cBioPortal divided into CNVs and mRNAs; (**B)** CanCord34 expression in Metabric breast cancer datasets. mRNA and CNV together, CNV alone and mRNA alone; and (**C**) Represents the amplification of a random chromosome region chr20:33490075-34540748, showing limited or albeit or no upregulation of a limited number of continuously organized protein-coding genes.

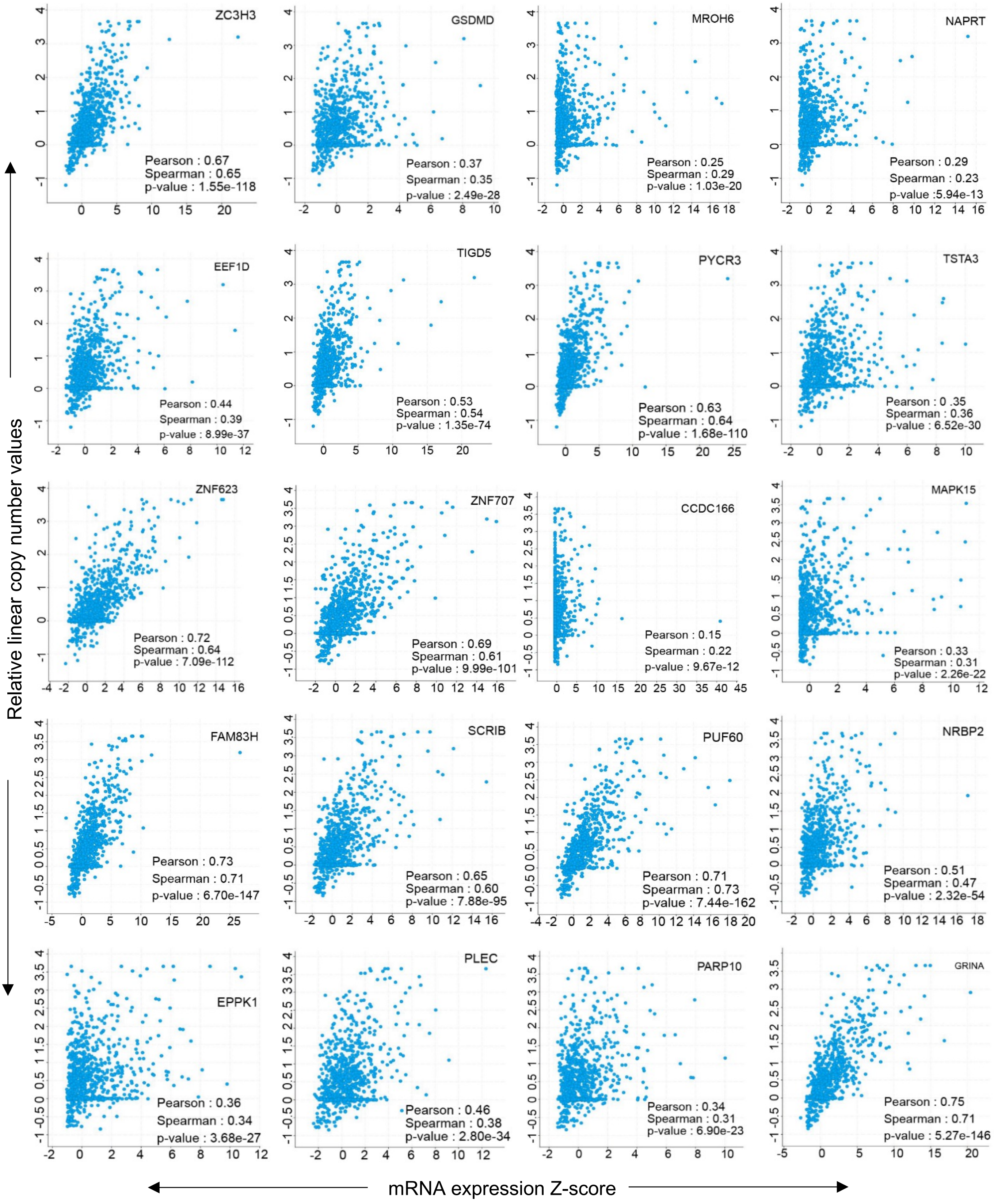

Supplementary Figure S5

Conti.

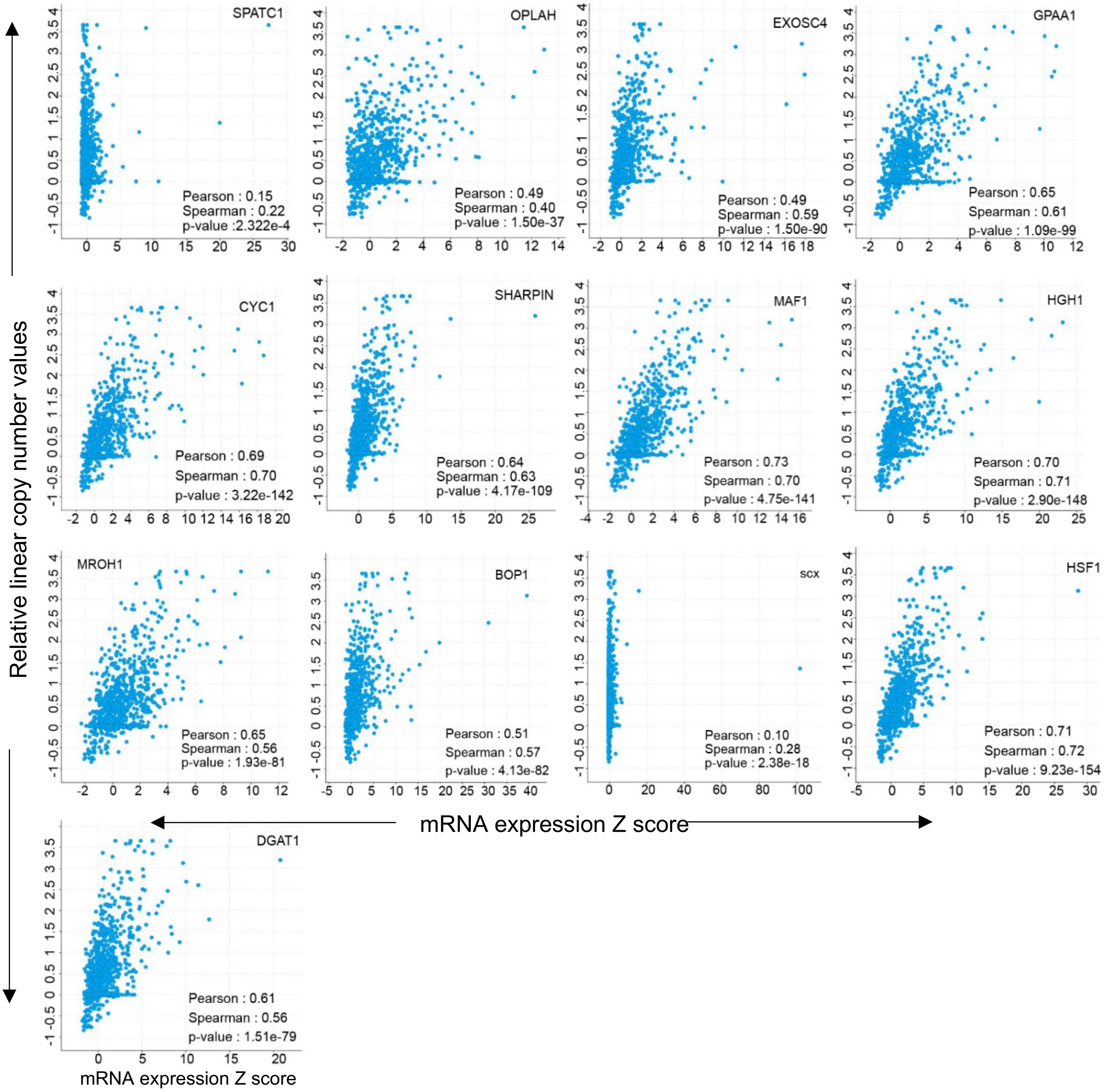

Supplementary Figure S5: Correlation between the mRNAs and CNVs of CanCord34 genes. Individual correlation between the mRNA and CNV of CanCord34 genes using the cBioportal dataset. X-axis, mRNA expression; Y-axis, relative copy number.

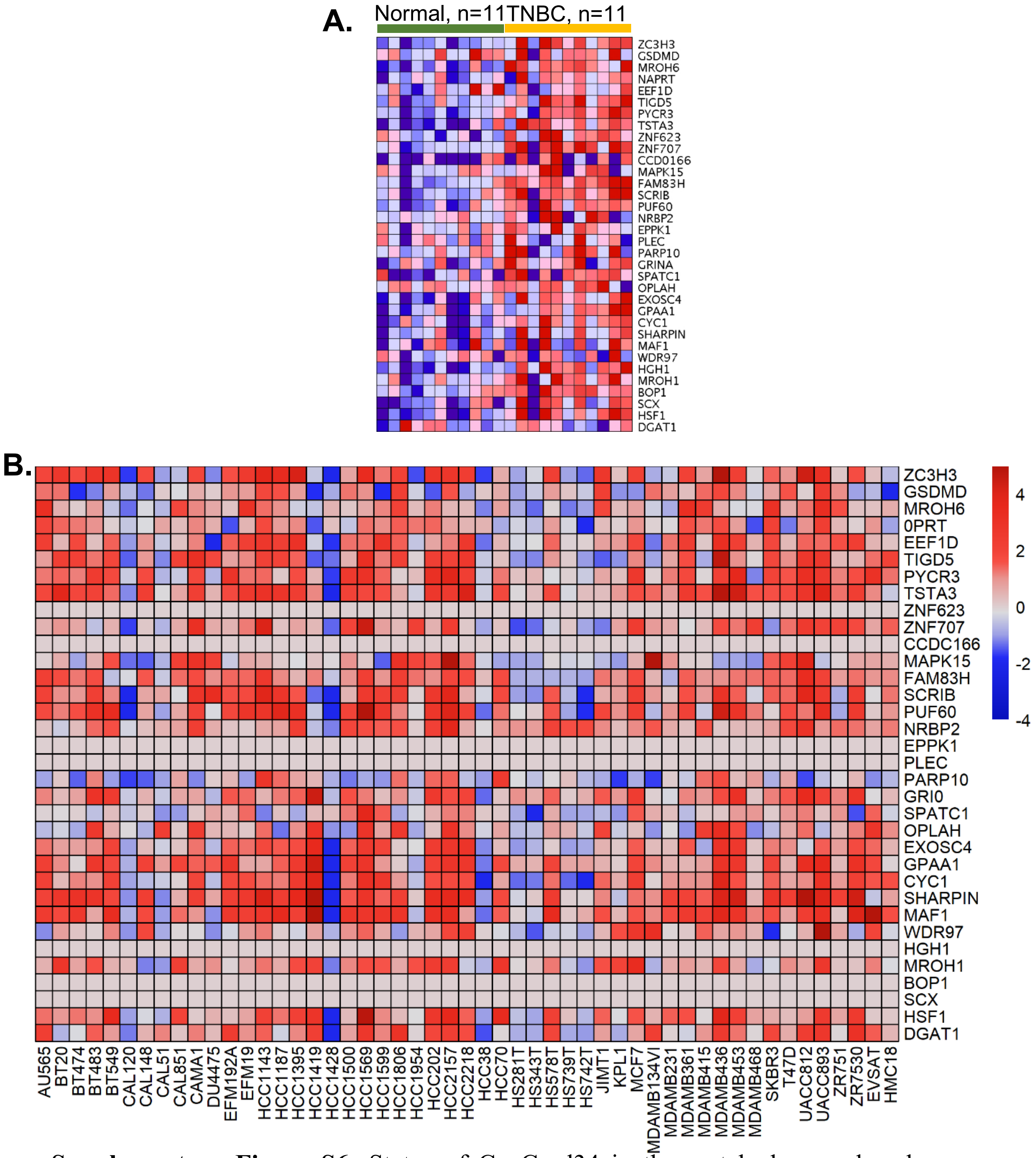

Supplementary Figure S6: Status of CanCord34 in the matched normal and breast cancer (**A**) and 51 breast cancer cell lines using the cBioportal CCLE dataset (**B**).

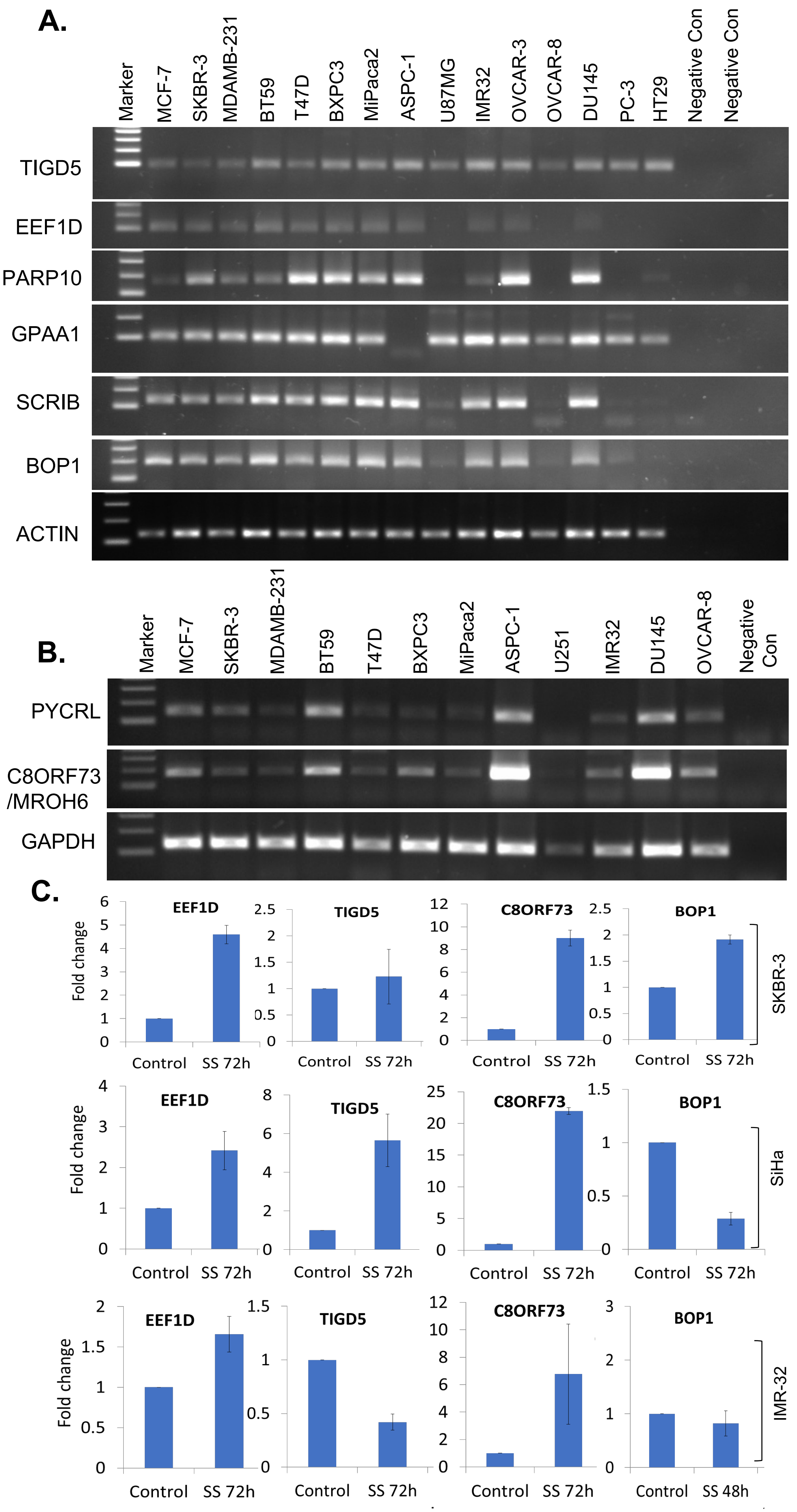

Supplementary Figure S7A. Expression of some CanCord34 gene family members in cancer cell lines. (**A-B)** RT-PCR expression of indicated genes in breast and other cancer cell lines; (C) Relative fold change expression of indicated genes in cancer cell lines. Con, control; SS 72, cells serum-starved for 72 hr.

TIGD5

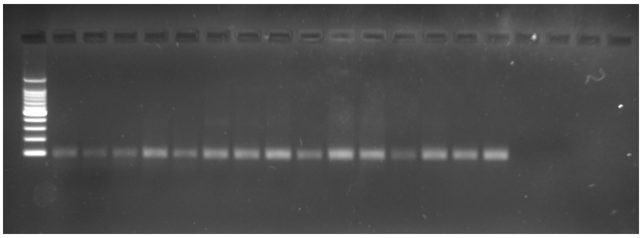

PARP10

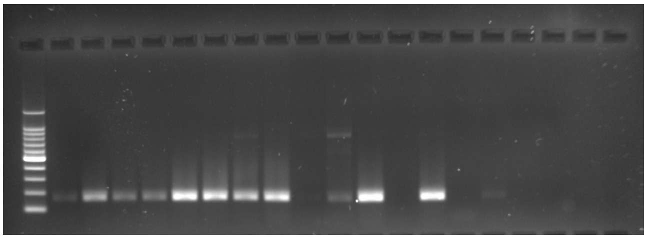

EEF1D

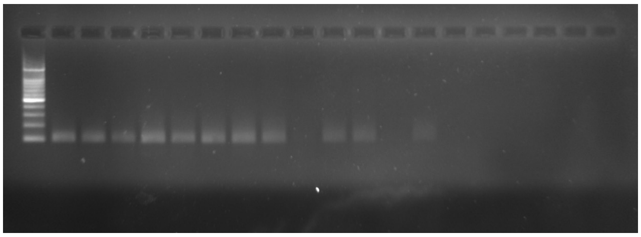

GPAA1

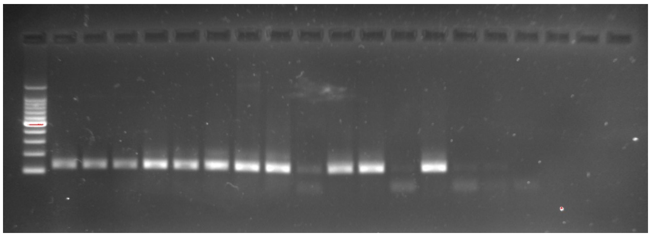

SCRIB

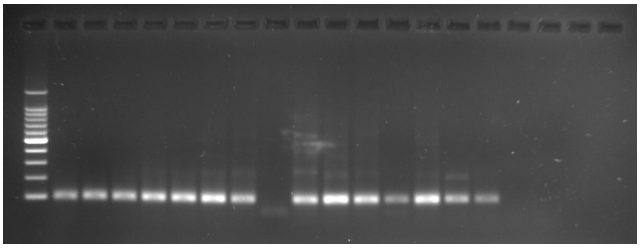

BOP1

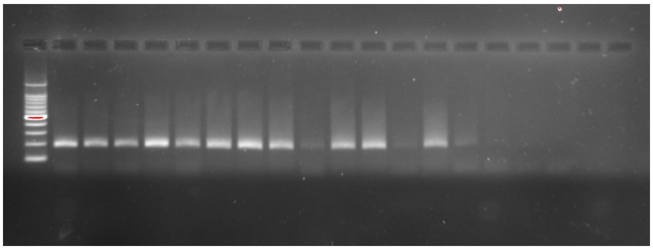

Actin

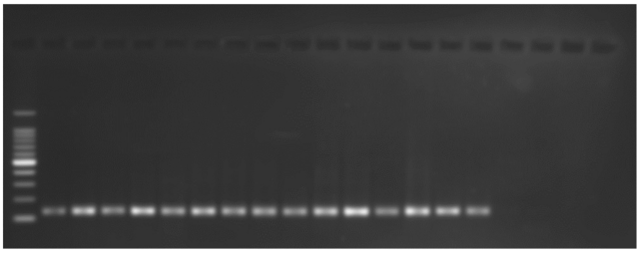

PYCRL

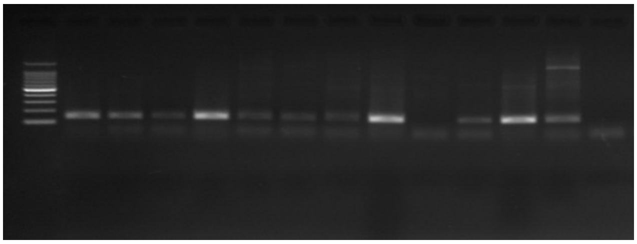

C8ORF73

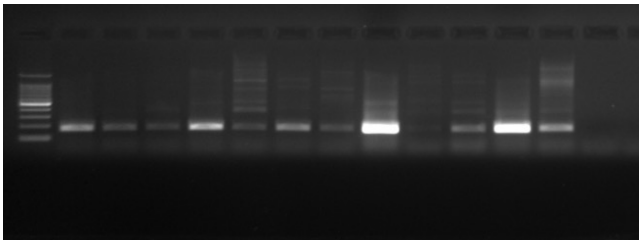

GAPDH

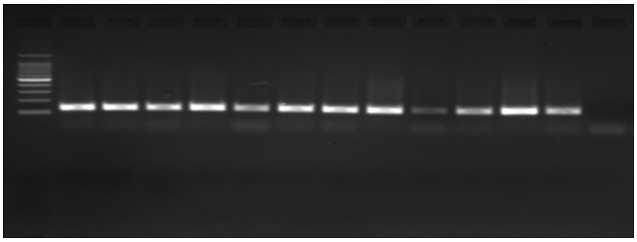

Supplementary Figure S7B: RT-PCR gel images of panels used in Supplementary Figure 7A and B.

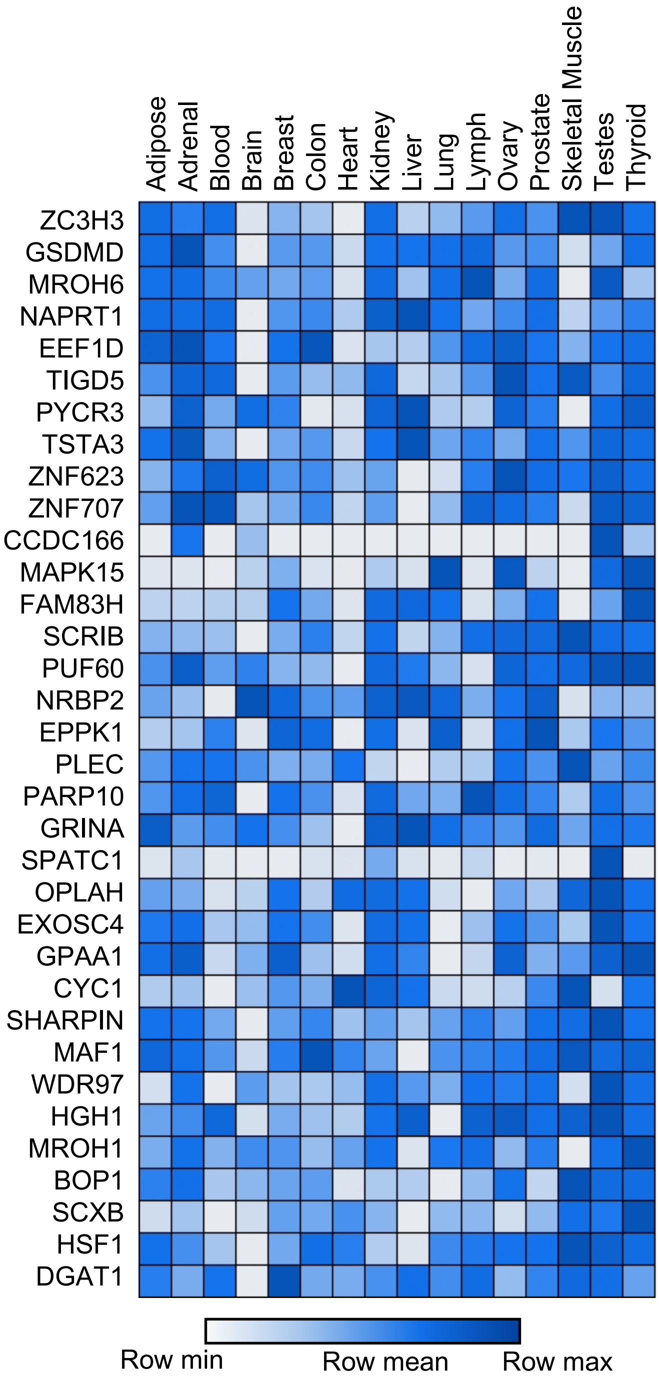

Supplementary Figure S8: CanCord34 expression in different clusters of normal tissues and mixture of tissues.

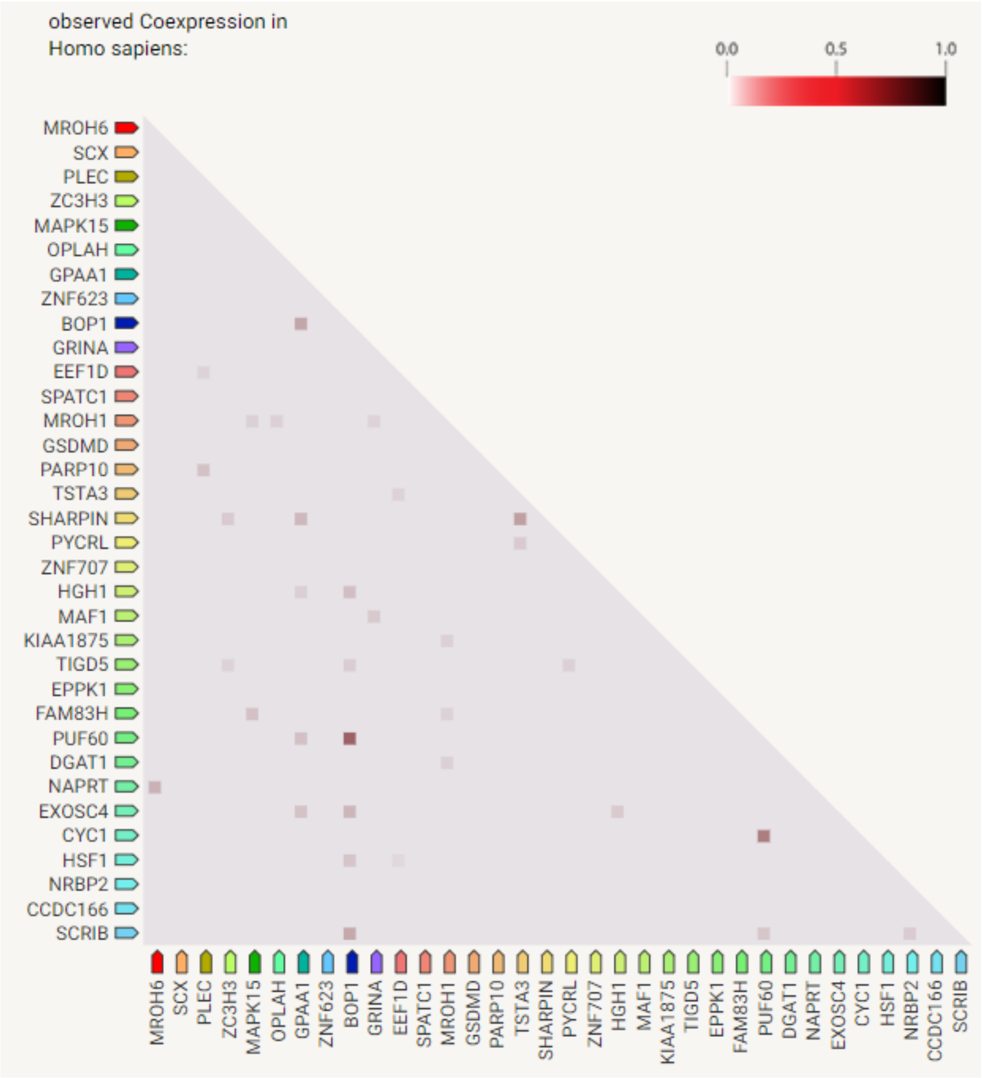

Supplementary Figure S9: Status of co-expression of CanCord34 genes in human tissues. The noticed positive correlation of the color intensity with the co-expression scores (Supplementary Table 1) based on the expression of mRNAs and proteins using the STRING program. The color codes in the x and y axis are meant to identify the genes.

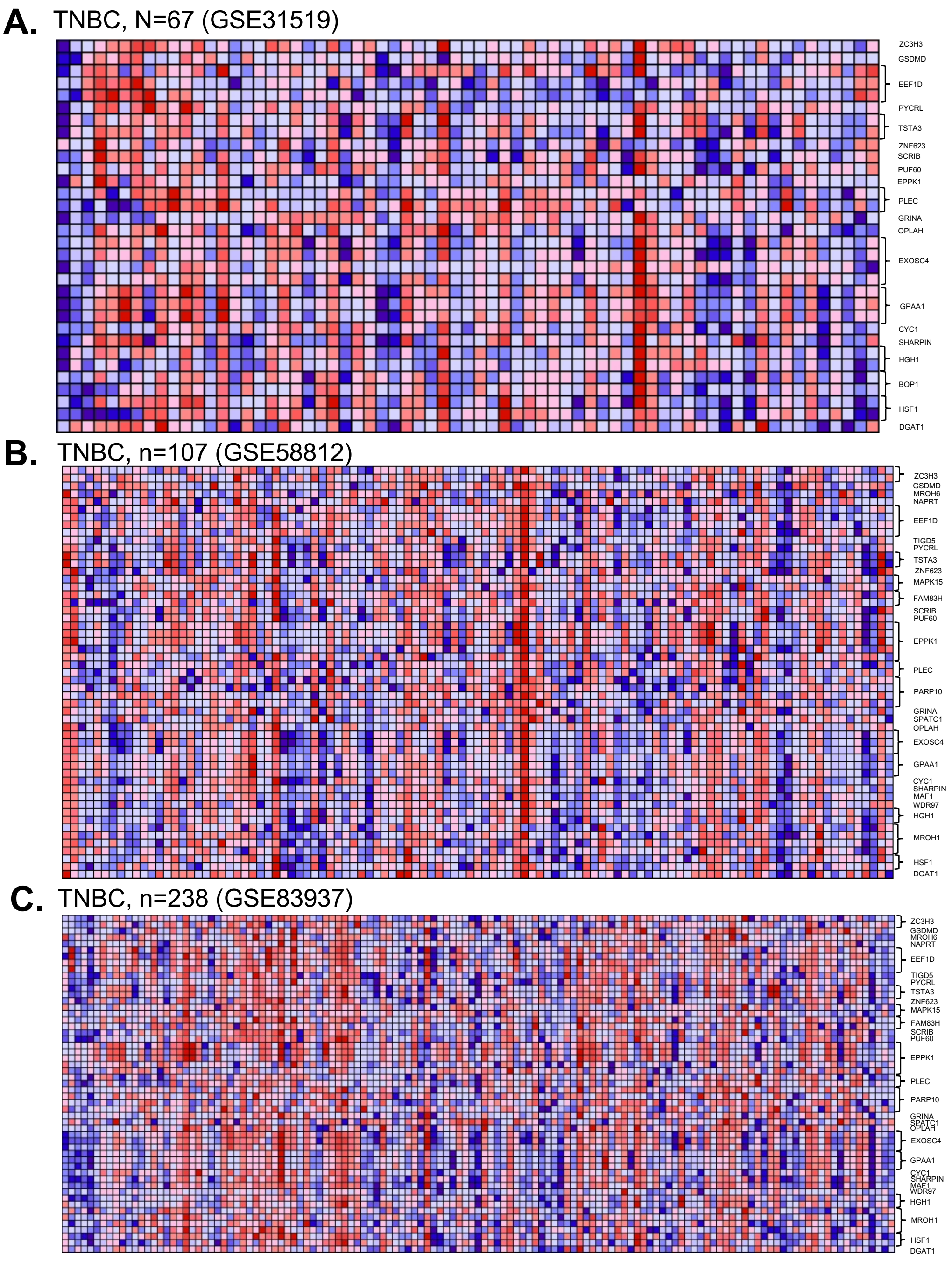

Supplementary Figure S10: Heatmap representation of CanCord34 genes in TNBC datasets. (**A)** GSE31519; **(B)** GSE58812; **(C)** GSE83937.

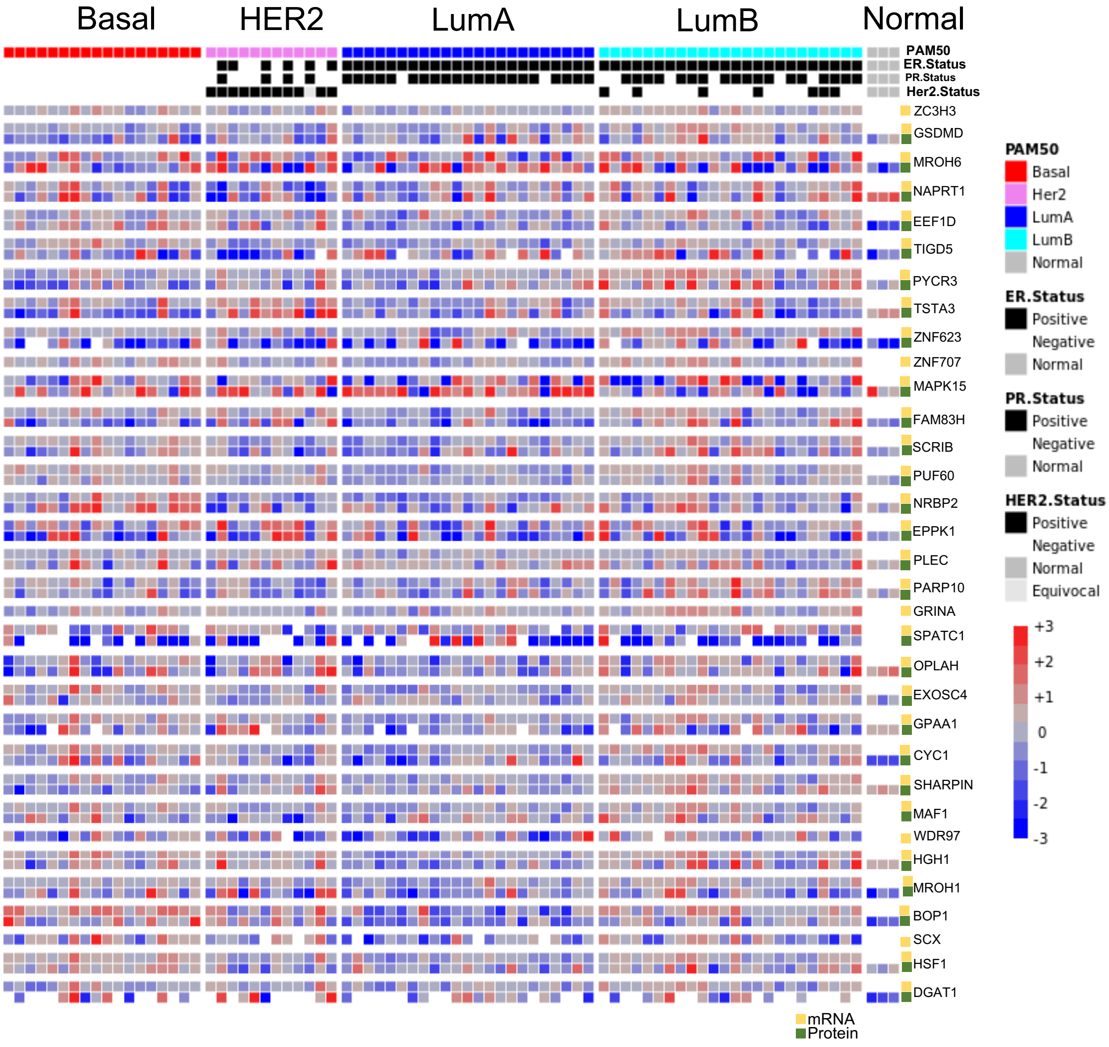

Supplementary Figure S11: Proteogenomic expression of CanCord34 genes in breast cancer dataset (37). The expression of CCDC166 was not detectable.

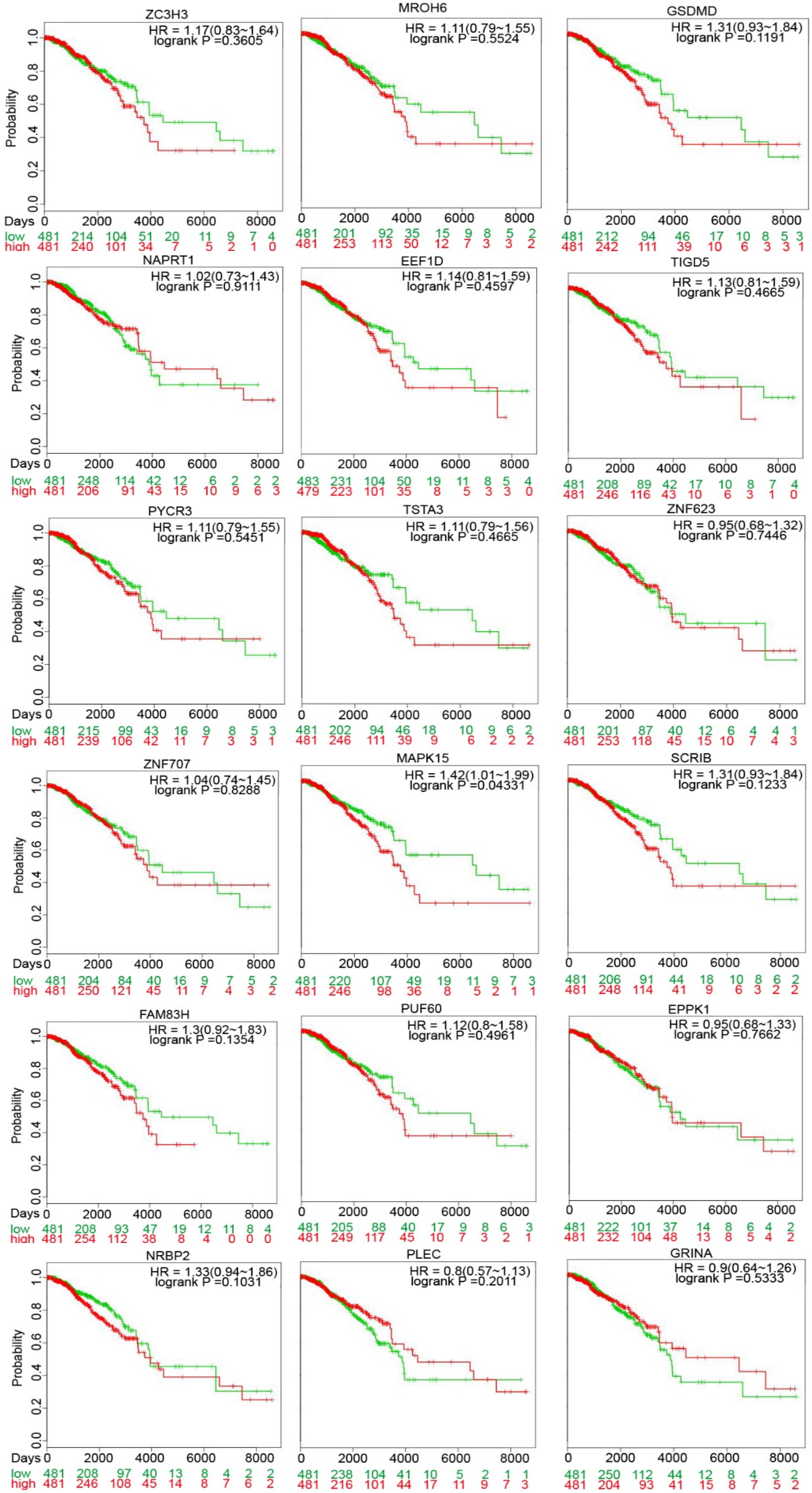

Supplementary Figure S12

Conti.

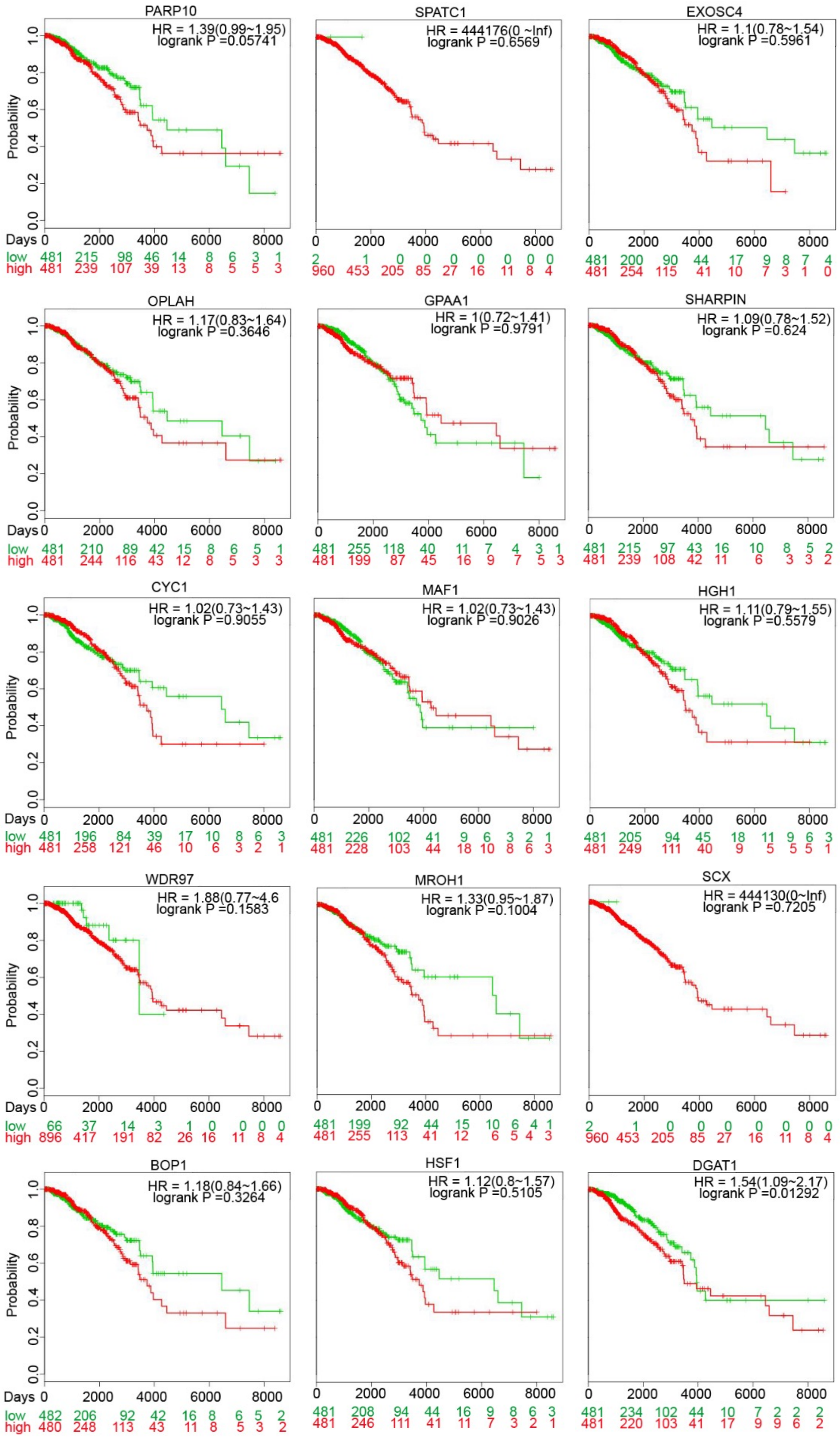

Supplementary Figure S12: The Kaplan–Meier survival plots of individual CanCord34 genes in breast cancer. The data represents univariant Kaplan-Meier curves for the low (green) and high (red) overexpressing risk groups, Hazard ratio and p-value of the survival curves are shown. The analysis was performed using the online SurvExpress platform.

	Total sample no	Total No of alteration in sequenced samples	No of gene altered
Esophageal Carcinoma	185	154 (83%) of 185	33
Liver Hepatocellular Carcinoma	373	304 (82%) of 373	33
Breast Invasive Carcinoma	1100	755 (69%) of 1100	32
Uveal Melanoma	80	73 (90%) of 80	31
Kidney Chromophobe	66	48 (73%) of 66	31
Lung Adenocarcinoma	517	369 (71%) of 517	31
Lung Squamous Cell Carcinoma	501	363 (72%) of 501	31
Ovarian Serous Cystadenocarcinoma	307	275 (90%) of 307	32
Prostate Adenocarcinoma	498	279 (56%) of 498	32
Skin Cutaneous Melanoma	472	279 (59%) of 472	31
Cholangiocarcinoma	36	25 (69%) of 36	31
Bladder Urothelial Carcinoma	408	297 (73%) of 408	31
Colorectal Adenocarcinoma	382	296 (78%) of 382	31
Cervical Squamous Cell Carcinoma and Endocervical Adenocarcinoma	306	202 (66%) of 306	30
Stomach Adenocarcinoma	415	320 (77%) of 415	31
Head and Neck Squamous Cell Carcinoma	522	392 (75%) of 522	31
Uterine Corpus Endometrial Carcinoma	177	118 (67%) of 177	31
Mesothelioma	87	59 (68%) of 87	30
Uterine Carcinosarcoma	57	52 (91%) of 57	29
Adrenocortical Carcinoma	79	57 (72%) of 79	29
Acute Myeloid Leukemia	173	93 (54%) of 173	29
Testicular Germ Cell Cancer	156	121 (78%) of 156	28
Pancreatic Adenocarcinoma	179	107 (60%) of 179	28
Brain Lower Grade Glioma	530	287 (56%) of 530	27
Kidney Renal Papillary Cell Carcinoma	291	153 (53%) of 291	27
Glioblastoma Multiforme	166	95 (59%) of 166	26
Kidney Renal Clear Cell Carcinoma	448	176 (39%) of 448	22
Thymoma	120	65 (54%) of 120	21
Lymphoid Neoplasm Diffuse Large B-cell Lymphoma	48	32 (67%) of 48	20
Pheochromocytoma and Paraganglioma	184	100 (56%) of 184	19
Sarcoma	263	138 (53%) of 263	18
Thyroid Carcinoma	509	239 (48%) of 509	18

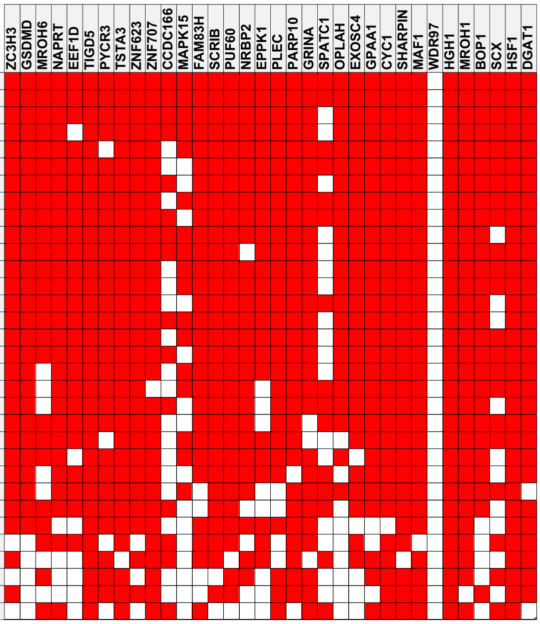

Supplementary Figure S13: Overview of alterations/upregulation of CanCord34 genes in human cancer with a minimum alteration rate of 5%. Red block, genes passed the minimum threshold; and white block, otherwise.

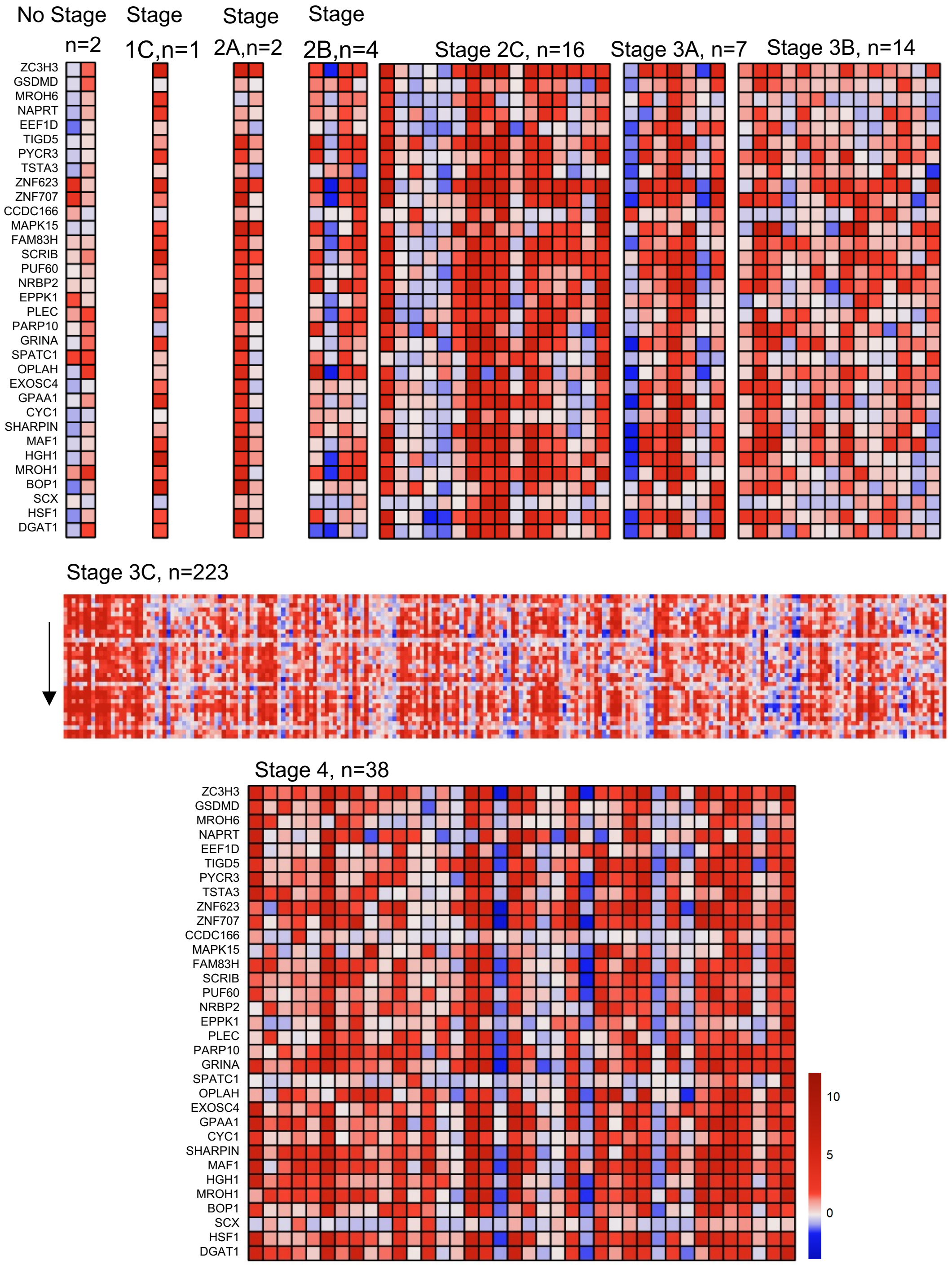

Supplementary Figure S14: Disease stage-wise representation of CanCord34 genes expression in ovarian cancer. The color represents the z-score of gene expression. WDR97 gene expression was absent in the database, and hence, eliminated from the representation here. The datasets were curated from the cBioportal database.

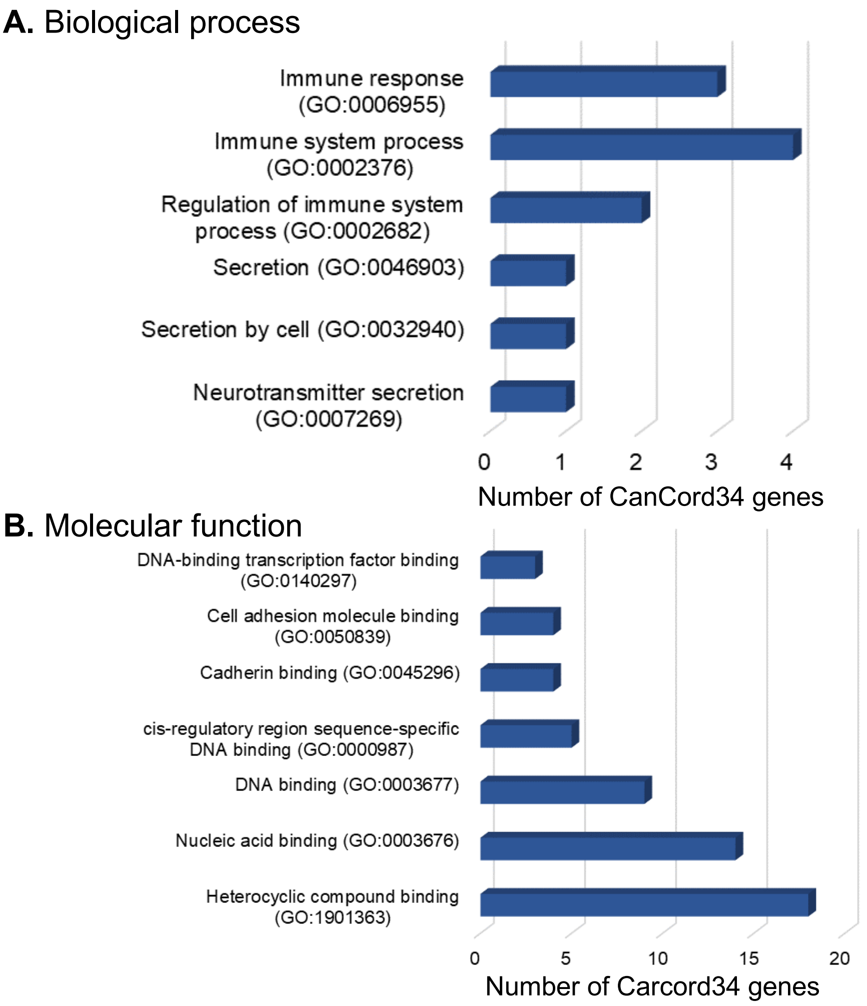

Supplementary Figure S15: Gene ontology enrichment analysis of CanCord34 genes. GO functional enrichment for CanCord34 genes were performed using Panther db. Significantly enriched top (**A**) Biological process and **(B)** Molecular functions are represented in separate bar charts.

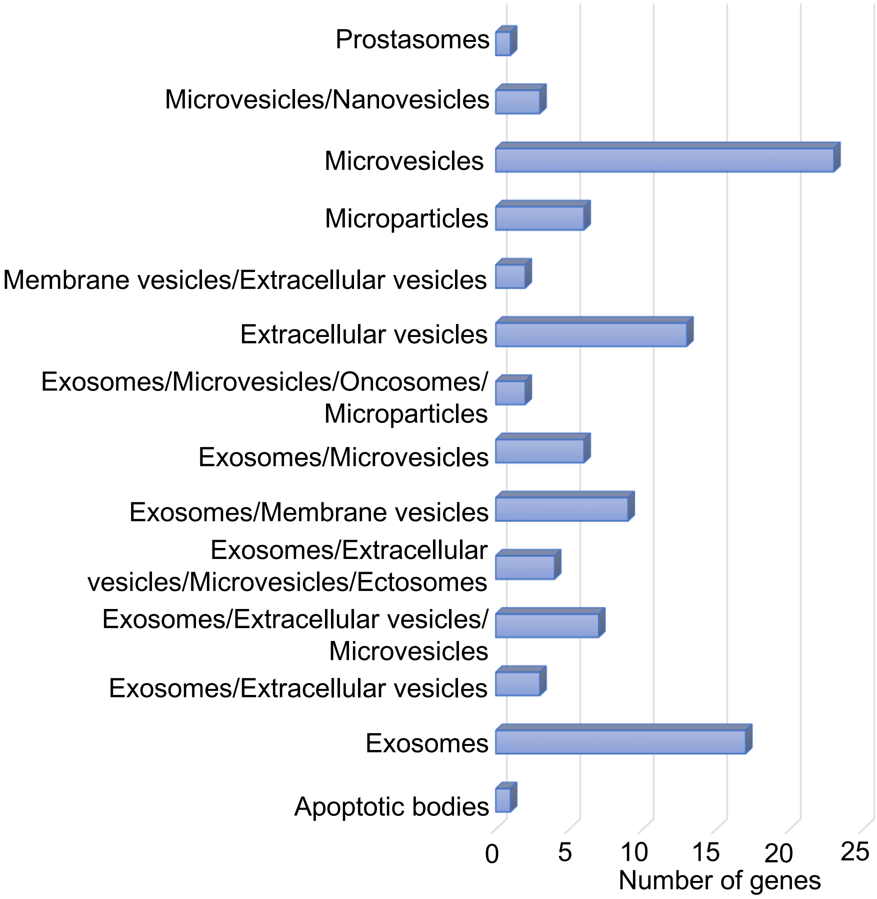

Supplementary Figure S16: CanCord34 genes as secretory molecules using the data retrieved from the VESICLEPEDIA.

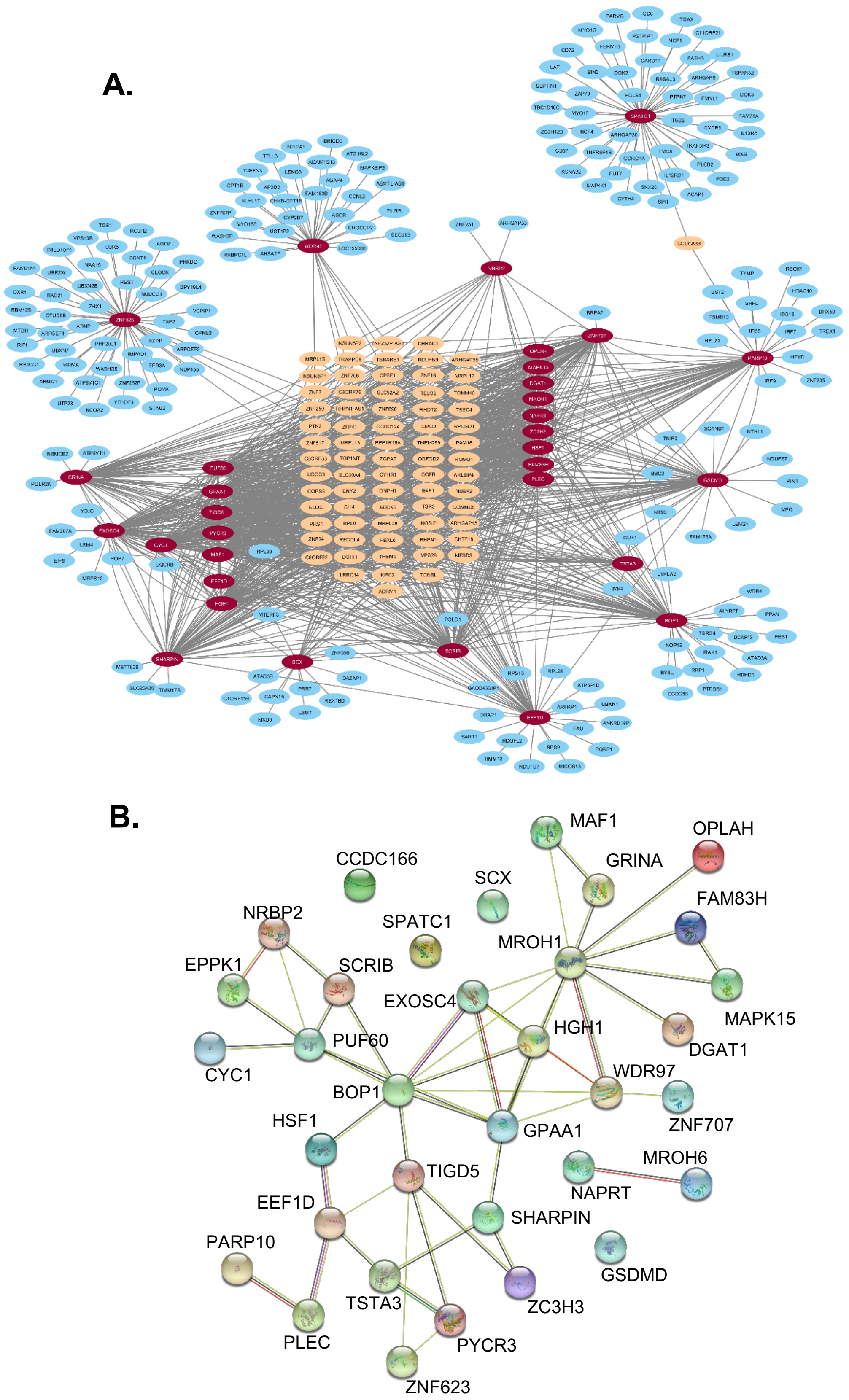

Supplementary Figure S17: Interconnection among CanCord genes in breast cancer. **(A)** Shared correlated genes among the CanCord34 are aligned towards the centre for the diagram; and CanCord34 genes are depicted in brown red. The top correlated genes with a r-value of >0.5 were considered for data presented. **(B)** Functional protein-protein interaction using the STRING online program.

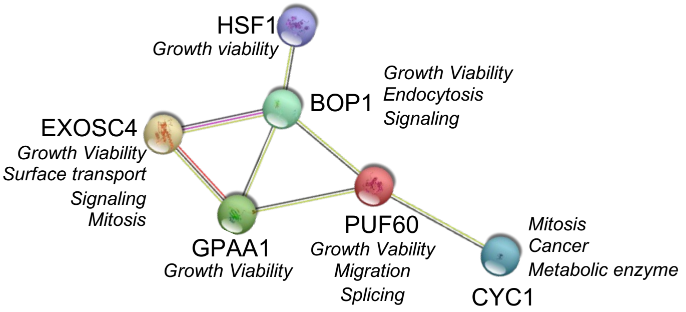

Supplementary Figure S18: Curated functions from Genome RNAi database (51) and evidence of protein-protein from STRING analysis of six of CanCord34 genes which has shown potential role in the cell viability.

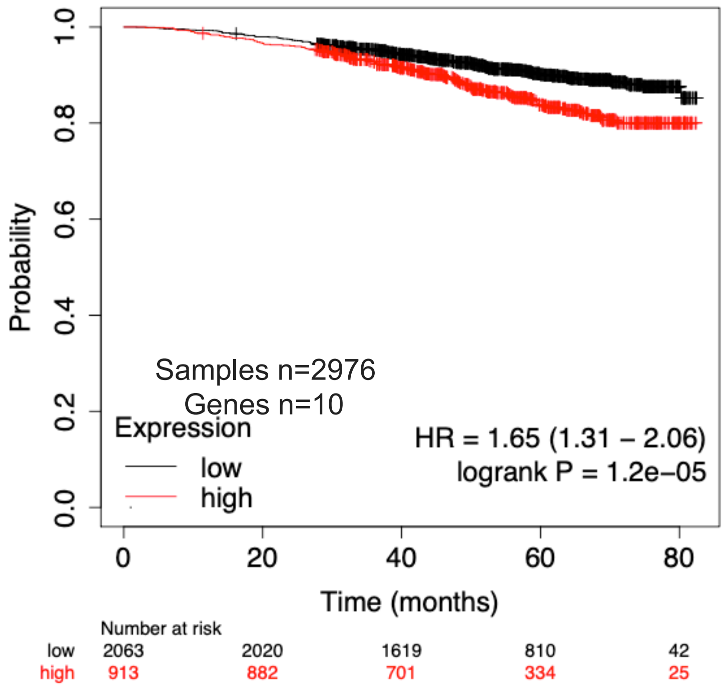

Supplementary Figure S19: The Kaplan–Meier survival plots of 10 secreted genes in breast cancer. The data represents multivariant Kaplan-Meier curves of 10 CanCord34 genes present in the secreted MVs. These genes include, PLEC. GSDMD, SCRIB, EXOSC4, CYC1, GPAA1, GRINA, HSF1, PUF60 and MAF1. Black and red curves represent the low and high expressing groups. The analysis was performed using an online KMplot platform.

Transcription Factors	CanCord34 genes																										Total number of shared CanCord34 genes
SPDEF	GPAA1	MAPK15	WDR97	SPATC1	MAF1	ZNF623	ZNF707	BOP1	SCRIB	DGAT1	MAFA	EPPK1	EEF1D	CCDC166	FAM83H	MROH6	NAPRT	NRBP2	OPLAH	PARP10	PLEC	PUF60	SHARPIN	TSTA3	ZC3H3	PYCR3	26
CTCF	CYC1	EXOSC4	HGH1	MROH1	GSDMD	GRINA	TIGD5	WDR97	SPATC1	MAF1	SCRIB	DGAT1	MAFA	EEF1D	FAM83H	MROH6	NAPRT	OPLAH	PARP10	PLEC	PUF60	SHARPIN	ZC3H3	PYCR3			24
ELF1	CYC1	EXOSC4	GPAA1	HSF1	GSDMD	TIGD5	WDR97	MAF1	ZNF623	ZNF707	BOP1	SCRIB	DGAT1	EPPK1	EEF1D	FAM83H	MROH6	NAPRT	PLEC	PUF60	SHARPIN	TSTA3	ZC3H3	PYCR3			24
MYC	EXOSC4	GPAA1	HGH1	MROH1	MAPK15	GSDMD	GRINA	TIGD5	SPATC1	ZNF623	ZNF707	BOP1	SCRIB	DGAT1	MAFA	EEF1D	FAM83H	MROH6	PARP10	PLEC	PUF60	TSTA3	PYCR3				23
EGR1	CYC1	GPAA1	MAPK15	GSDMD	GRINA	TIGD5	WDR97	MAF1	ZNF623	ZNF707	SCRIB	MAFA	EPPK1	EEF1D	FAM83H	NRBP2	PLEC	PUF60	SHARPIN	TSTA3	PYCR3						21
YY1	CYC1	GPAA1	MAPK15	GRINA	TIGD5	WDR97	MAF1	ZNF707	BOP1	DGAT1	MAFA	CCDC166	MROH6	NAPRT	NRBP2	OPLAH	PLEC	PUF60	SHARPIN	ZC3H3	PYCR3						21
PAX5	EXOSC4	GPAA1	HGH1	MROH1	GSDMD	GRINA	TIGD5	SPATC1	ZNF623	ZNF707	BOP1	SCRIB	NAPRT	PARP10	PLEC	PUF60	SHARPIN	TSTA3	ZC3H3	PYCR3							20
SNAI2	CYC1	GPAA1	HGH1	MAPK15	SCX	GSDMD	WDR97	MAF1	SCRIB	DGAT1	EPPK1	CCDC166	FAM83H	MROH6	NAPRT	NRBP2	PLEC	SHARPIN	TSTA3	PYCR3							20
SP1	CYC1	GPAA1	HGH1	MROH1	MAPK15	GRINA	TIGD5	MAF1	SCRIB	DGAT1	MAFA	EPPK1	MROH6	NAPRT	NRBP2	PLEC	PUF60	TSTA3	ZC3H3	PYCR3							20
KLF5	CYC1	EXOSC4	HSF1	GSDMD	GRINA	TIGD5	MAF1	ZNF623	ZNF707	BOP1	SCRIB	EPPK1	FAM83H	NAPRT	NRBP2	PLEC	PUF60	TSTA3	PYCR3								19
RELA	CYC1	EXOSC4	GPAA1	MAPK15	GSDMD	GRINA	TIGD5	SPATC1	MAF1	ZNF623	ZNF707	SCRIB	DGAT1	MAFA	NRBP2	PARP10	PLEC	PUF60	TSTA3								19
ZNF552	CYC1	HGH1	MROH1	MAPK15	SCX	GRINA	TIGD5	WDR97	BOP1	SCRIB	MAFA	EPPK1	CCDC166	FAM83H	MROH6	NRBP2	PUF60	SHARPIN	PYCR3								19
GABPA	EXOSC4	GPAA1	HGH1	HSF1	GSDMD	TIGD5	MAF1	ZNF623	ZNF707	SCRIB	DGAT1	MAFA	FAM83H	NAPRT	PLEC	PUF60	TSTA3	ZC3H3									18
ZBTB7A	CYC1	GPAA1	MAPK15	GSDMD	GRINA	TIGD5	MAF1	ZNF623	DGAT1	MAFA	EPPK1	EEF1D	FAM83H	NRBP2	PLEC	PUF60	ZC3H3	PYCR3									18
AR	CYC1	EXOSC4	GPAA1	SCX	GSDMD	WDR97	SPATC1	MAF1	EEF1D	MROH6	NAPRT	OPLAH	PARP10	SHARPIN	ZC3H3	PYCR3											16
ERG	EXOSC4	GPAA1	HGH1	HSF1	MAF1	ZNF623	ZNF707	BOP1	SCRIB	DGAT1	EPPK1	FAM83H	MROH6	NAPRT	PUF60	TSTA3											16
ETS1	CYC1	EXOSC4	GPAA1	GSDMD	TIGD5	MAF1	ZNF623	ZNF707	SCRIB	DGAT1	EPPK1	FAM83H	MROH6	NAPRT	PUF60	TSTA3											16
IRF1	CYC1	MAPK15	GSDMD	GRINA	TIGD5	MAF1	DGAT1	MAFA	EPPK1	FAM83H	MROH6	NRBP2	PLEC	PUF60	TSTA3	PYCR3											16
MYOD1	MAPK15	GSDMD	TIGD5	WDR97	MAF1	DGAT1	MAFA	EEF1D	CCDC166	MROH6	NAPRT	NRBP2	OPLAH	SHARPIN	TSTA3	PYCR3											16
RCOR1	EXOSC4	GPAA1	GSDMD	GRINA	SPATC1	ZNF707	SCRIB	DGAT1	MAFA	EPPK1	FAM83H	MROH6	PARP10	PLEC	TSTA3	PYCR3											16
TCF12	CYC1	GPAA1	MROH1	GSDMD	TIGD5	WDR97	MAF1	DGAT1	MAFA	EPPK1	FAM83H	NAPRT	NRBP2	SHARPIN	TSTA3	PYCR3											16
ELK1	EXOSC4	GPAA1	HSF1	GSDMD	TIGD5	MAF1	ZNF623	ZNF707	BOP1	SCRIB	EPPK1	FAM83H	PUF60	TSTA3	PYCR3												15
FOXP1	CYC1	EXOSC4	ZNF623	FAM83H	CYC1	EXOSC4	GPAA1	HSF1	GRINA	ZNF623	ZNF707	SCRIB	DGAT1	PUF60	TSTA3												15
KDM5B	HSF1	MAPK15	GRINA	WDR97	SPATC1	BOP1	SCRIB	DGAT1	MAFA	EEF1D	CCDC166	NAPRT	NRBP2	PLEC	SHARPIN												15
KLF4	HSF1	MAPK15	GRINA	TIGD5	MAF1	BOP1	SCRIB	MAFA	EEF1D	FAM83H	MROH6	PLEC	PUF60	ZC3H3	PYCR3												15
TFAP2C	MAPK15	GRINA	TIGD5	SPATC1	MAF1	ZNF623	ZNF707	SCRIB	DGAT1	MAFA	EEF1D	MROH6	NRBP2	PARP10	PLEC												15
BCLAF1	CYC1	EXOSC4	GPAA1	GSDMD	SPATC1	MAF1	ZNF623	ZNF707	SCRIB	PARP10	PLEC	PUF60	TSTA3	ZC3H3													14
E2F1	CYC1	GPAA1	HSF1	TIGD5	MAF1	ZNF623	BOP1	DGAT1	MAFA	EPPK1	FAM83H	NAPRT	NRBP2	TSTA3													14
MTA3	EXOSC4	GPAA1	HSF1	GSDMD	SPATC1	MAF1	ZNF623	ZNF707	SCRIB	DGAT1	PARP10	PUF60	TSTA3	PYCR3													14
MAX	CYC1	EXOSC4	GPAA1	HSF1	MAPK15	GSDMD	TIGD5	BOP1	SCRIB	MAFA	OPLAH	PLEC	PYCR3														13
TBP	GSDMD	GRINA	TIGD5	SPATC1	MAF1	ZNF707	MAFA	EPPK1	FAM83H	PARP10	PUF60	ZC3H3	PYCR3														13
ETV1	EXOSC4	GRINA	ZNF623	ZNF707	MAFA	EPPK1	NAPRT	NRBP2	OPLAH	PUF60	TSTA3	ZC3H3															12
KLF9	CYC1	MAPK15	TIGD5	SCRIB	MAFA	EPPK1	FAM83H	MROH6	NRBP2	PUF60	TSTA3	PYCR3															12
MYBL2	EXOSC4	GPAA1	HSF1	GSDMD	MAF1	ZNF623	ZNF707	BOP1	NAPRT	PUF60	TSTA3	PYCR3															12
STAT5A	GPAA1	GSDMD	SPATC1	MAF1	ZNF623	ZNF707	SCRIB	NAPRT	PARP10	PUF60	TSTA3	ZC3H3															12
BCL6	CYC1	EXOSC4	GSDMD	TIGD5	SPATC1	ZNF623	MAFA	PARP10	PLEC	PUF60	TSTA3																11
CDX2	CYC1	GPAA1	HSF1	MAPK15	WDR97	MAF1	SCRIB	MROH6	NAPRT	SHARPIN	TSTA3																11
TP53	CYC1	SPATC1	MAF1	ZNF623	BOP1	EPPK1	EEF1D	FAM83H	NRBP2	PARP10	TSTA3																11
ARNT	CYC1	EXOSC4	MAPK15	GRINA	MAF1	SCRIB	MAFA	OPLAH	PUF60	ZC3H3																	10
ATF2	EXOSC4	SPATC1	MAF1	ZNF623	SCRIB	DGAT1	PARP10	PUF60	TSTA3	ZC3H3																	10
CREB1	CYC1	HSF1	GRINA	TIGD5	MAF1	BOP1	DGAT1	PUF60	ZC3H3	PYCR3																	10
ELK4	EXOSC4	GPAA1	MAF1	ZNF623	ZNF707	EPPK1	FAM83H	MROH6	PUF60	TSTA3																	10
FOXD2	CYC1	EXOSC4	GSDMD	MAF1	FAM83H	NAPRT	NRBP2	PUF60	TSTA3	PYCR3																	10
GMEB2	EXOSC4	GPAA1	HSF1	TIGD5	ZNF623	BOP1	SCRIB	FAM83H	PUF60	TSTA3																	10
KDM5A	EXOSC4	GPAA1	GRINA	WDR97	NAPRT	PLEC	PUF60	SHARPIN	ZC3H3	PYCR3																	10
NR2F1	MAPK15	GSDMD	EXOSC4	MROH1	WDR97	SPATC1	MAF1	CCDC166	PARP10	SHARPIN																	10
NRF1	GPAA1	MROH1	GRINA	TIGD5	MAF1	BOP1	DGAT1	MAFA	TSTA3	ZC3H3																	10
SMAD4	HGH1	TIGD5	MAF1	DGAT1	MAFA	MROH6	NAPRT	PLEC	PUF60	ZC3H3																	10
SRF	TIGD5	WDR97	DGAT1	MAFA	EEF1D	PLEC	PUF60	SHARPIN	PYCR3																		10
STAT3	GPAA1	TIGD5	SPATC1	ZNF623	ZNF707	SCRIB	DGAT1	PARP10	TSTA3	ZC3H3																	10
ZEB1	GPAA1	GSDMD	GRINA	SPATC1	ZNF707	SCRIB	FAM83H	NAPRT	PARP10	PLEC																	10
E2F6	GPAA1	HSF1	TIGD5	BOP1	SCRIB	MAFA	EPPK1	PLEC	PYCR3																		9
SP4	MAF1	BOP1	SCRIB	DGAT1	MAFA	PUF60	TSTA3	PYCR3																			9
TCF4	TIGD5	SPATC1	SCRIB	MAFA	EEF1D	FAM83H	PARP10	PUF60	TSTA3																		9
BHLHE40	HSF1	GSDMD	TIGD5	BOP1	DGAT1	MAFA	PLEC	PUF60																			8
ESR1	HSF1	GSDMD	MAF1	BOP1	SCRIB	FAM83H	MROH6	NAPRT																			8
FOS	GPAA1	MAPK15	SPATC1	MAF1	ZNF623	FAM83H	PARP10	PYCR3																			8
CEBPB	MAPK15	SPATC1	MAF1	PARP10	TSTA3	ZC3H3																					6

Supplementary Figure S20: The transcription factor motifs present in the Cancord34 genes within 1kb up- and down-stream of TSS . The genes aligned in the positive and negative strands are shown as blue and red colors, respectively. The numbers of shared genes for TFs in a row are shown to the right. TFs are manually curated from ChiPBase v2.0 database.

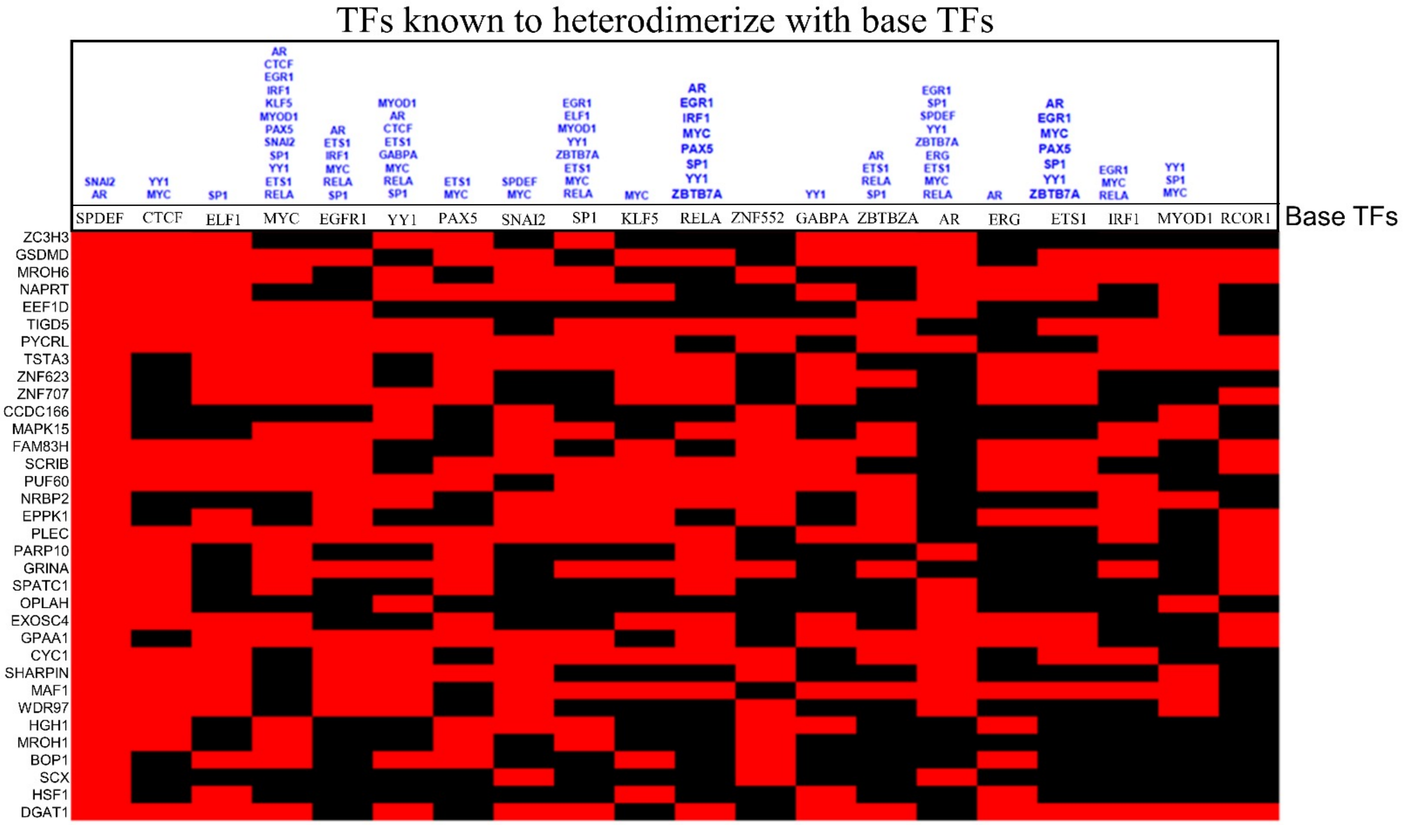

Supplementary Figure S21: Transcription factors binding motifs in CanCord34 genes and their heterodimerization with other TFs.

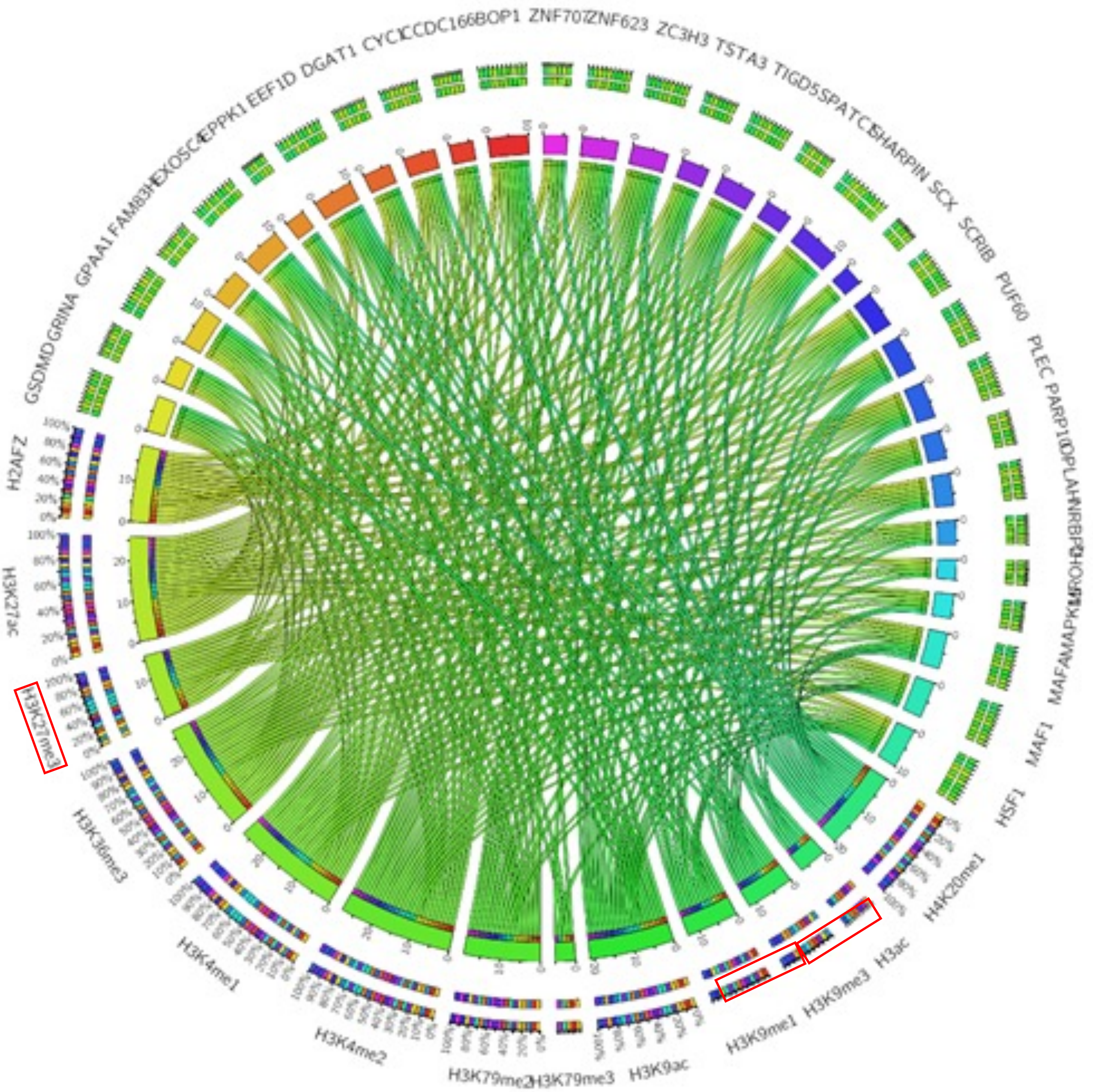

Supplementary Figure S22: Circos plot showing various activated and repressive histone marks shared across the CanCord34 genes. The repressive marks are marked in red boxes. The data were curated from the ENCODE epigenome database and plotted using the online Circos tools.

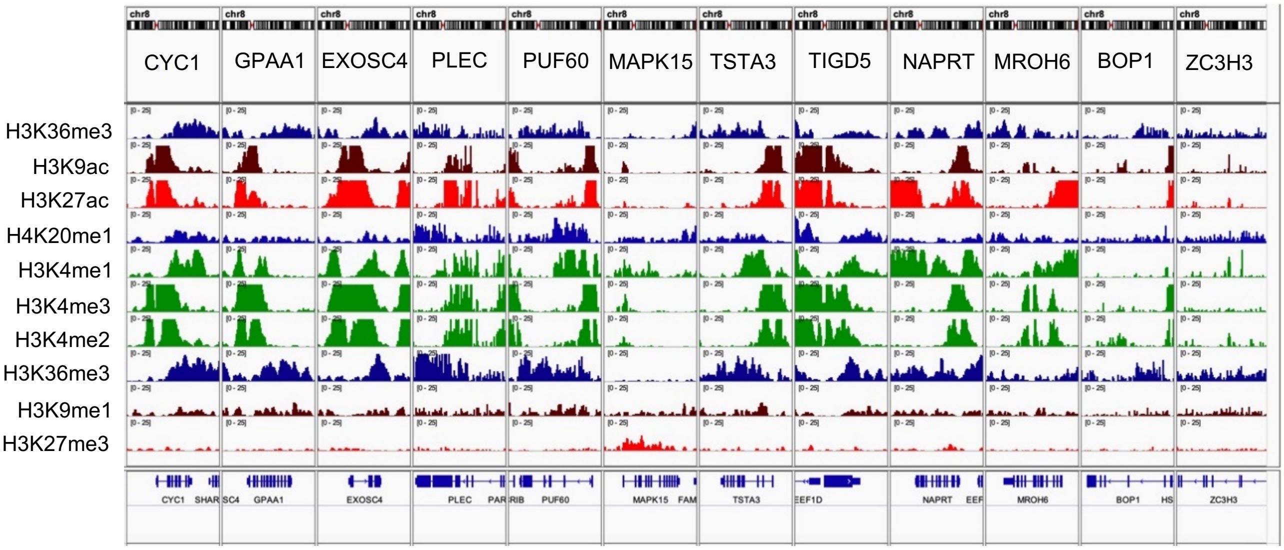

Supplementary Figure S23. Examples of shared TF's and histone marks across the genomic coordinates of a few members of CanCord34 genes using the UCSC genome browser.

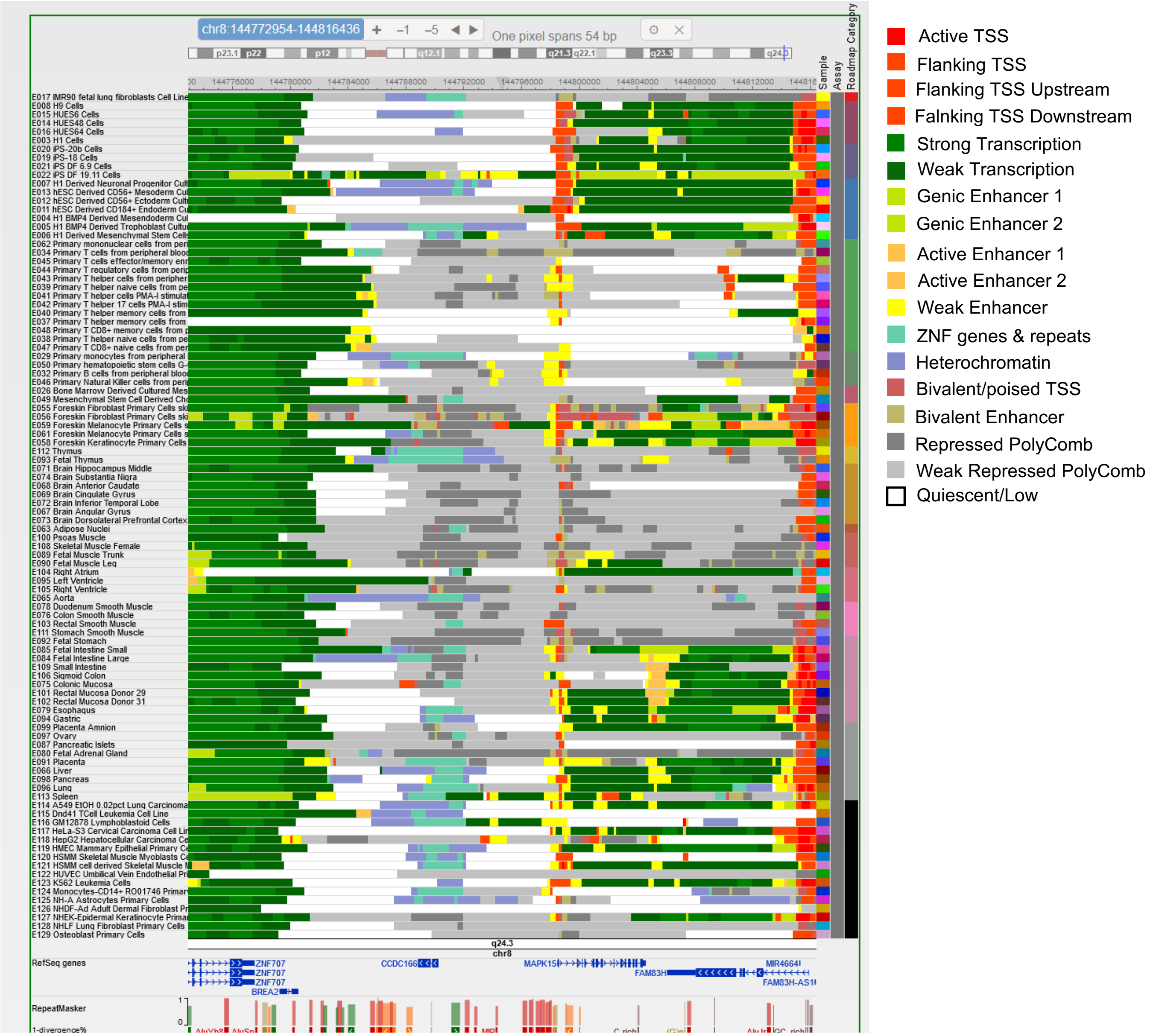

Supplementary Figure S24: The genomic coordinates of CanCord 34 genes. The genomic coordinates and possible regulatory elements were visualized using the UCSC genome browser in different cell lines. The regulatory elements are labelled with distinct codes.

A.

GO biological process complete	Homo sapiens - REFLIST (20595)	upload_1 (34)	upload_1 (expected)	upload_1 (over/under)	upload_1 (fold Enrichment)	upload_1 (raw P-value)	upload_1 (FDR)
immune response (GO:0006955)	1678	3	2.77	+	1.08	7.54E-01	1.00E+00
immune system process (GO:0002376)	2370	4	3.91	+	1.02	1.00E+00	1.00E+00
regulation of immune system process (GO:0002682)	1418	2	2.34	-	0.85	1.00E+00	1.00E+00
secretion (GO:0046903)	486	1	0.8	+	1.25	5.56E-01	1.00E+00
secretion by cell (GO:0032940)	366	1	0.6	+	1.66	4.57E-01	1.00E+00
neurotransmitter secretion (GO:0007269)	78	1	0.13	+	7.77	1.22E-01	1.00E+00

B.

GO molecular function complete	Homo sapiens - REFLIST (20595)	upload_1 (34)	upload_1 (expected)	upload_1 (over/under)	upload_1 (fold Enrichment)	upload_1 (raw P-value)	upload_1 (FDR)
DNA-binding transcription factor binding (GO:0140297)	402	3	0.66	+	4.52	2.87E-02	1.00E+00
cell adhesion molecule binding (GO:0050839)	537	4	0.89	+	4.51	1.16E-02	1.00E+00
cadherin binding (GO:0045296)	323	4	0.53	+	7.5	1.98E-03	1.00E+00
cis-regulatory region sequence-specific DNA binding (GO:00	1223	5	2.02	+	2.48	4.91E-02	1.00E+00
DNA binding (GO:0003677)	2494	9	4.12	+	2.19	1.74E-02	1.00E+00
nucleic acid binding (GO:0003676)	3989	14	6.59	+	2.13	3.51E-03	1.00E+00
heterocyclic compound binding (GO:1901363)	5945	18	9.81	+	1.83	3.72E-03	1.00E+00

Supplementary Table S1: Selected Biological process (A) and molecular function with p-values (B).
